# Microbial Biostimulants for Drought Resilience in Plants: Mechanisms, Plant–Microbiome Interactions, and Sustainable Agricultural Applications

**DOI:** 10.3390/plants15193022

**Published:** 2026-10-02

**Authors:** Younes Rezaee Danesh, José M. Mulet, Rosa Porcel

**Affiliations:** 1 Department of Plant Protection, Faculty of Agriculture, Van Yüzüncü Yıl University, 65090 Van, Türkiye; 2 Instituto de Biología Molecular y Celular de Plantas, Universitat Politècnica de València-Consejo Superior de Investigaciones Científicas, 46022 Valencia, Spain; roporrol@upv.es

**Keywords:** microbial biostimulants, drought resilience, plant–microbiome interactions, sustainable agriculture, abiotic stress, soil health

## Abstract

Drought stress is one of the most significant challenges for global agriculture, adversely affecting crop yields and food security. In recent years, microbial biostimulants have emerged as a promising solution to enhance plant resilience against such abiotic stresses. This review explores the mechanisms by which microbial biostimulants, including beneficial bacteria and fungi, confer drought tolerance in plants. Key interactions between plants and their associated microbiomes are highlighted, emphasizing the roles of root-associated microbes in promoting plant growth, nutrient uptake, and stress mitigation. We examine microbial-induced plant responses, including improved water relations, photosynthetic performance, and osmotic adjustment. Additionally, the review examines the sustainability of microbial biostimulant use in agricultural practices, addressing their potential to reduce reliance on chemical inputs and improve soil health. Evidence from field studies across diverse crops is also discussed, highlighting the potential of microbial biostimulants to improve plant performance and yield stability under water-limited conditions while emphasizing the context-dependent nature of their efficacy. Furthermore, we underscore the importance of microbial diversity and community composition in maximizing the benefits of biostimulants. By integrating microbial biostimulants into conventional agricultural practices, farmers can enhance drought resilience, thereby contributing to sustainable food production systems. This review aims to provide a comprehensive understanding of the interactions between microbial biostimulants and plants under drought stress, and to highlight future research directions for optimizing their use in sustainable agriculture.

## 1. Introduction

Drought is an increasingly significant limiting factor for global agricultural crop productivity, leading to shorter crop-growing seasons and substantial yield losses as a direct consequence of ongoing climate change. In light of this pressing challenge, the development and application of microbial biostimulants that enhance plant drought resilience have emerged as a promising, environmentally sustainable strategy for modern agriculture [1,2]. Studies focusing on plant–microbe interactions have revealed that diverse microbial communities associated with plant organs, including roots, leaves, and flowers, play fundamental roles in improving plant growth, physiological performance, and stress adaptation under drought conditions [3]. Both individual microbial strains and microbial consortia can alleviate drought-induced stress. Consortia, in which multiple beneficial microorganisms may function complementarily or synergistically, can combine several plant-beneficial traits; however, their advantage over single-strain inoculants is not universal and depends on the specific host–microbe combination, environmental conditions, and response variable evaluated [4]. The mechanisms underlying the drought-protective effects of microbial biostimulants involve several interconnected physiological and molecular processes, including enhanced water uptake efficiency, improved osmotic adjustment through the accumulation of compatible solutes, modulation of phytohormone biosynthesis and signaling pathways, stimulation of nutrient acquisition through plant–microbe interactions, and activation of antioxidant defense systems. Furthermore, microbial biostimulants regulate root exudation patterns, thereby promoting the establishment of beneficial rhizosphere microbiomes that further strengthen plant adaptation to water-deficit conditions [5]. These multifaceted mechanisms can collectively improve plant fitness and crop productivity under drought stress, although the magnitude of these effects varies with the inoculant, host plant, and environment.

Microbial biostimulants that confer drought resilience offer numerous advantages for sustainable agricultural production. By improving water-use efficiency, these beneficial microorganisms help utilize limited water resources more effectively while stabilizing crop productivity under increasingly unpredictable climatic conditions. Moreover, microbial biostimulants can be tailored to individual agroecosystems by formulating native microbial consortia or optimized plant–microbe combinations that maximize plant performance and environmental adaptation [6]. Several field studies have confirmed observations obtained under controlled conditions, demonstrating improvements in root architecture, root activity, nutrient uptake, and drought tolerance following microbial inoculation, although field responses are generally more variable. Nevertheless, significant knowledge gaps remain regarding the precise molecular mechanisms governing plant–microbe interactions under drought stress, the consistency of microbial performance across different soil types and climatic conditions, and the long-term stability of introduced microbial communities [7,8]. Long-term, multi-location field experiments are therefore needed to evaluate plant performance, yield stability, soil health, and microbiome dynamics under realistic agricultural conditions. In addition, greater standardization of experimental protocols, microbial formulations, and reporting criteria is essential to improve reproducibility and facilitate meaningful comparisons among studies. Addressing these research priorities will substantially enhance our understanding of drought-alleviating microbial biostimulants and accelerate their successful transition from laboratory research to large-scale agricultural applications, thereby supporting sustainable crop production under increasing water scarcity and climate change [9].

Despite the rapid expansion of research on microbial biostimulants and their role in plant drought tolerance, current knowledge remains fragmented across different microbial groups, plant species, and experimental systems. Most previous reviews have focused on individual categories of beneficial microorganisms, such as plant growth-promoting rhizobacteria (PGPR), arbuscular mycorrhizal fungi (AMF), or endophytic microorganisms, or have emphasized specific physiological responses without providing an integrated understanding of the complex mechanisms governing microbe-induced drought resilience. Moreover, recent advances in microbiome engineering, synthetic microbial consortia, multi-omics technologies, and precision agriculture have substantially expanded our understanding of plant–microbe interactions under drought stress. Yet, these advances have not yet been comprehensively synthesized. Therefore, this review aims to provide an up-to-date, integrated overview of microbial biostimulants involved in alleviating drought stress by critically examining their physiological, biochemical, molecular, and microbiome-mediated mechanisms. Furthermore, this review discusses recent advances in microbial consortium design, formulation strategies, field applications, and commercialization challenges while identifying current knowledge gaps and future research priorities for developing resilient and sustainable crop production systems under increasingly water-limited environments [8,10].

This narrative review was developed through a structured literature search using Web of Science, Scopus, PubMed, and Google Scholar. The search focused primarily on peer-reviewed literature published between 2015 and 2026, while earlier seminal studies were included where necessary to provide conceptual or mechanistic context. Search terms were used individually and in combination and included “microbial biostimulants,” “plant growth-promoting rhizobacteria,” “PGPR,” “arbuscular mycorrhizal fungi,” “AMF,” “*Trichoderma*,” “endophytes,” “microbial consortia,” “synthetic microbial communities,” “drought stress,” “drought tolerance,” “water-use efficiency,” “osmotic adjustment,” “antioxidant defense,” “plant–microbiome interactions,” and “drought resilience.” Studies were prioritized when they provided experimental or mechanistic evidence concerning microbial-mediated plant responses to water deficit, plant–microbiome interactions, field performance, formulation and delivery, or agricultural applications. Peer-reviewed original research articles and relevant review articles were considered, whereas studies unrelated to plant drought responses or lacking direct relevance to microbial biostimulant-mediated stress alleviation were excluded. Because the objective was to provide an integrative narrative synthesis rather than a systematic review or meta-analysis, study selection was guided by relevance to the thematic scope of the review rather than by a formal systematic-review protocol.

## 2. Mechanisms of Microbial Biostimulants in Drought Resilience

Microbial biostimulants enhance plant drought resilience through multiple interconnected physiological, biochemical, and molecular mechanisms. These include improved water acquisition and water-use efficiency, modulation of phytohormone and stress-signaling networks, enhanced osmotic adjustment and membrane stability, reinforcement of antioxidant defense and reactive oxygen species homeostasis, and improved nutrient acquisition and nutrient-use efficiency. Rather than functioning independently, these responses are closely coordinated and collectively enable plants to maintain cellular homeostasis, photosynthetic performance, growth, and productivity under water-deficit conditions. The major mechanisms underlying microbial-mediated drought resilience are discussed in the following subsections.

### 2.1. Enhancement of Water Uptake and Use Efficiency

**Figure 1:**
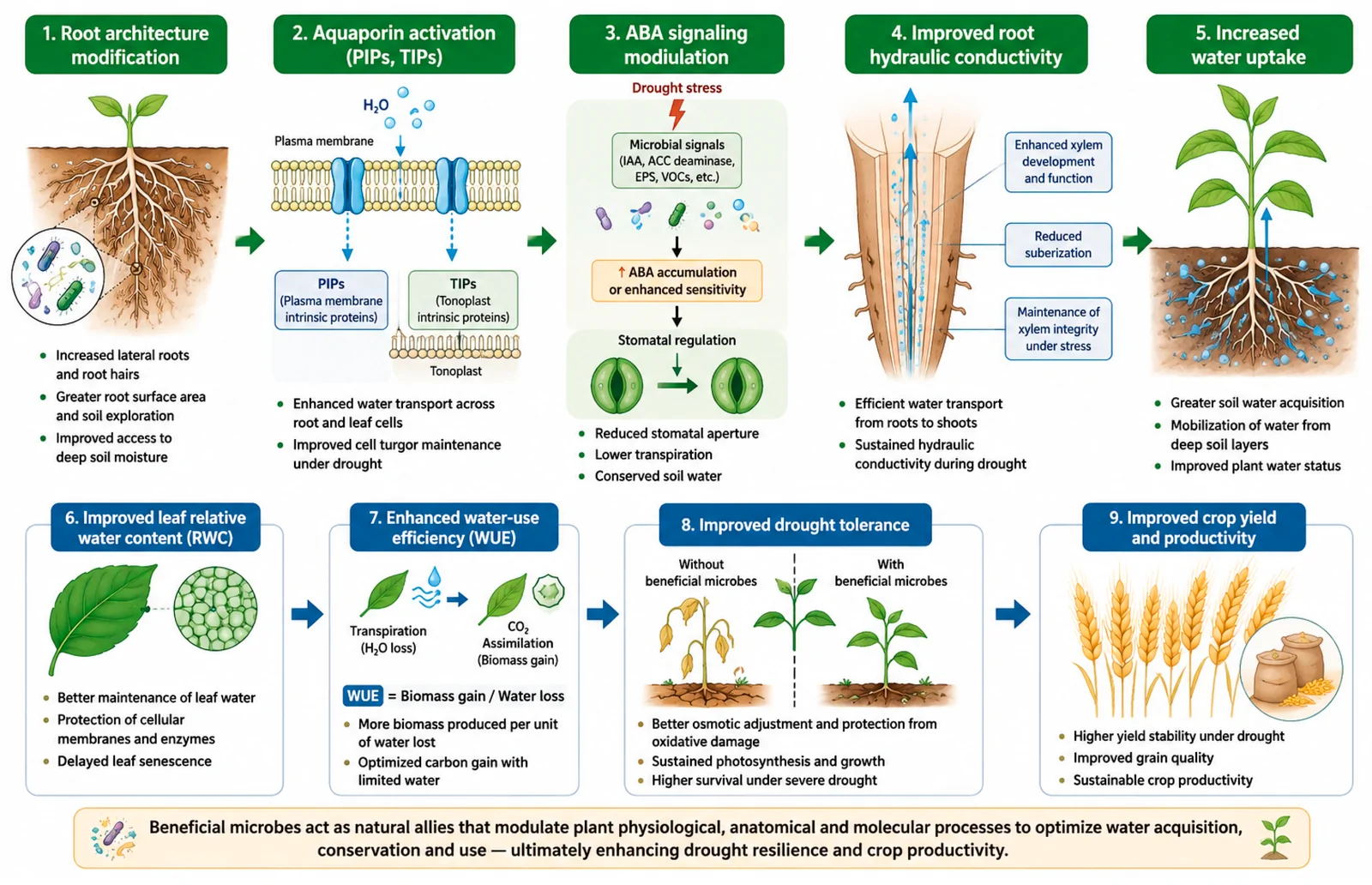
Microbial mechanisms influencing plant water relations and water-use efficiency under drought stress. Beneficial microorganisms improve plant adaptation to water deficit through complementary structural, physiological, and molecular mechanisms. Microbial inoculation promotes root elongation, lateral root formation, root hair development, and root biomass, thereby increasing soil exploration and access to available water. Microorganisms can also enhance root hydraulic conductivity and regulate aquaporins, including plasma membrane intrinsic proteins (PIPs) and tonoplast intrinsic proteins (TIPs), facilitating water transport across cellular membranes. These responses contribute to improved plant water status, maintenance of leaf relative water content (RWC), stomatal regulation, photosynthetic performance, and biomass accumulation under drought conditions. Improvements in water-use efficiency (WUE), however, depend on the metric evaluated and may include intrinsic WUE (net CO_2_ assimilation/stomatal conductance; A/g_s_), instantaneous transpiration efficiency (net CO_2_ assimilation/transpiration rate; A/E), whole-plant WUE (biomass produced relative to cumulative water use), or field-scale water productivity based on crop yield relative to water input. Thus, changes in RWC, hydraulic properties, or photosynthetic performance are considered indicators of improved plant water relations rather than direct measurements of WUE unless an appropriate water-use metric is quantified. In the figure, large arrows indicate the proposed sequential progression from microbe-induced changes in root architecture and physiological regulation to improved plant water relations, drought tolerance, and crop productivity, whereas internal arrows indicate the direction of water transport or regulatory/functional relationships within individual processes. Upward arrows (↑) indicate an increase or enhancement in the corresponding process or response.

Soil moisture availability is one of the most critical environmental factors governing plant growth, development, and agricultural productivity. However, the increasing frequency and intensity of drought events associated with global climate change have made water scarcity one of the greatest constraints on sustainable crop production worldwide. Consequently, improving water-use efficiency (WUE) has become a major objective for developing climate-resilient agricultural systems. Among the various biological approaches, microbial biostimulants have emerged as an effective strategy for enhancing plant water acquisition and utilization through complementary structural and physiological mechanisms [11,12]. Water-use efficiency is a scale-dependent concept, and its interpretation depends on the metric employed [13,14]. At the leaf level, intrinsic water-use efficiency (iWUE) is commonly expressed as the ratio of net CO_2_ assimilation (A) to stomatal conductance (g_s_), whereas instantaneous transpiration efficiency is expressed as the ratio of CO_2_ assimilation to transpiration rate (A/E) [13,14]. At the whole-plant level, WUE generally describes biomass accumulation relative to cumulative water use or transpiration. Under field conditions, agronomic WUE or irrigation water productivity is typically evaluated as crop yield relative to total water supply or irrigation water applied, respectively. Because the studies considered in this review used different experimental systems and measurement approaches, the term WUE is used only when supported by a reported water-use metric; changes in relative water content, stomatal behavior, root hydraulic properties, or photosynthetic performance are otherwise described as improvements in plant water relations rather than as direct measurements of WUE. As mentioned before, plant-associated microorganisms stimulate root elongation, lateral root formation, root hair development, and root biomass, thereby increasing the soil volume explored by roots and improving access to available soil moisture. These structural modifications are often accompanied by enhanced root hydraulic conductivity and regulation of aquaporins, particularly plasma membrane intrinsic proteins (PIPs) and tonoplast intrinsic proteins (TIPs), which facilitate water transport across cellular membranes. Microbial inoculation can also influence ABA-responsive aquaporins and dehydration-responsive proteins, thereby improving root water permeability, leaf relative water content, and whole-plant hydraulic conductance under water deficit [2,15,16,17]. Collectively, microbial-mediated modifications of root architecture and water-transport processes can enable inoculated plants to maintain higher tissue water status, sustain photosynthetic activity, improve stomatal regulation, and preserve biomass accumulation under drought stress [18,19]. Where water consumption, transpiration, gas-exchange parameters, or yield relative to water input have been quantified, these responses may also translate into improvements in the corresponding WUE metric. The principal physiological and molecular mechanisms through which microbial biostimulants enhance plant water-use efficiency under drought stress are summarized in Figure 1. These interconnected processes collectively improve plant water status, water-use efficiency, and drought resilience.

### 2.2. Hormonal Modulation and Stress Signaling

One of the principal mechanisms by which microbial biostimulants influence plant drought responses is the modulation of phytohormone homeostasis and stress-signaling networks. These effects can be distinguished at three interconnected mechanistic levels: direct microbial production or metabolism of hormone-related compounds, microbe-induced modulation of endogenous plant hormone homeostasis, and downstream activation or modification of host signaling pathways. Distinguishing these levels is important because the magnitude and direction of hormonal responses can vary with plant species or genotype, microbial strain, drought intensity and duration, and the developmental or stress stage at which the interaction is evaluated [17,20,21]. At the microbial level, beneficial microorganisms may directly produce phytohormones or hormone-like compounds, particularly indole-3-acetic acid (IAA), cytokinins, and gibberellins, or alter hormone precursor availability. ACC deaminase-producing microorganisms represent a distinct mechanism because they metabolize 1-aminocyclopropane-1-carboxylate (ACC), thereby modifying the substrate available for plant ethylene biosynthesis. These direct microbial activities can influence root architecture, root growth, and other developmental processes that affect plant responses to declining water availability [17,20,21].

At the host level, microbial inoculation can modify endogenous hormone biosynthesis, degradation, transport, sensitivity, and homeostasis rather than supplying the hormone directly. This is particularly important for abscisic acid (ABA), a central regulator of plant drought responses. Microbe-induced changes in ABA homeostasis and signaling can influence stomatal closure, transpirational water loss, aquaporin activity, osmotic adjustment, and expression of drought-responsive genes. Microbial effects on auxin, cytokinin, gibberellin, jasmonic acid (JA), salicylic acid (SA), brassinosteroid (BR), strigolactone (SL), and ethylene pathways can further modify the balance between growth and stress acclimation under water deficit [17,20,21]. Downstream of these hormonal changes, host signaling networks integrate hormonal signals with Ca^2+^-dependent signaling, reactive oxygen species (ROS), nitric oxide (NO), and mitogen-activated protein kinase (MAPK) cascades. These signaling processes regulate stress-responsive transcription factors, including DREB, NAC, WRKY, MYB, bZIP, and MYC families, thereby coordinating transcriptional responses associated with antioxidant defense, osmotic adjustment, water transport, and cellular protection during drought [2,22,23,24]. Importantly, these responses are context-dependent; the same microbial taxon or hormonal pathway may produce different physiological outcomes depending on the crop genotype, microbial strain, drought severity and duration, and timing of inoculation relative to stress development.

**Figure 2:**
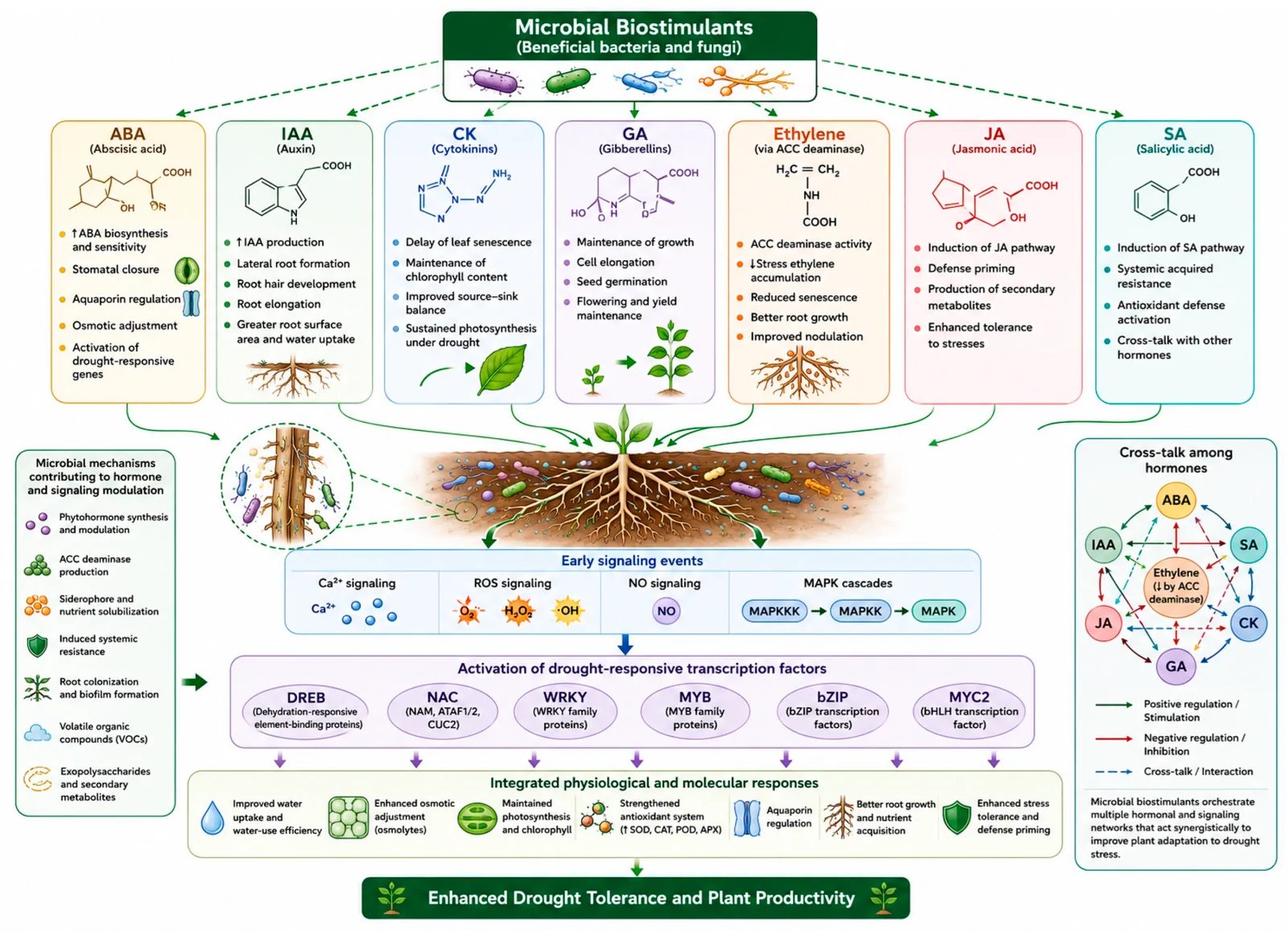
Microbial modulation of phytohormone homeostasis and stress-signaling networks under drought stress. Beneficial microbial biostimulants, including bacteria and fungi, influence plant drought responses through interconnected microbial, hormonal, signaling, and physiological mechanisms. Direct microbial activities include phytohormone synthesis and modulation, ACC deaminase activity, siderophore production and nutrient solubilization, induced systemic resistance, root colonization and biofilm formation, production of volatile organic compounds (VOCs), and exopolysaccharides and secondary metabolites. Microbial effects on phytohormone homeostasis involve abscisic acid (ABA), indole-3-acetic acid (IAA), cytokinins (CK), gibberellins (GA), ethylene, jasmonic acid (JA), and salicylic acid (SA). In particular, microbial production or modulation of IAA, CK, and GA can influence root development, growth, photosynthesis, and source–sink relationships, whereas modulation of ABA contributes to stomatal regulation, aquaporin activity, osmotic adjustment, and drought-responsive gene expression. ACC deaminase-mediated degradation of 1-aminocyclopropane-1-carboxylate (ACC) can reduce excessive stress-induced ethylene accumulation. JA and SA pathways contribute to defense priming, antioxidant responses, systemic resistance, and interactions with other hormonal pathways. These hormonal pathways form an interconnected cross-talk network rather than acting independently. Microbial and hormonal signals are further integrated with early signaling events involving Ca^2+^-dependent signaling, reactive oxygen species (ROS), nitric oxide (NO), and mitogen-activated protein kinase (MAPK) cascades, leading to activation of drought-responsive transcription factors, including DREB, NAC, WRKY, MYB, bZIP, and MYC2. Downstream responses include improved water uptake and water-use efficiency, enhanced osmotic adjustment, maintenance of photosynthesis and chlorophyll, strengthened antioxidant defense, aquaporin regulation, improved root growth and nutrient acquisition, and enhanced stress tolerance and defense priming, collectively contributing to increased drought tolerance and plant productivity. In the hormone cross-talk network, green arrows indicate positive regulation or stimulation, red arrows indicate negative regulation or inhibition, and blue dashed arrows indicate cross-talk or interaction; other directional arrows indicate the proposed flow of microbial, hormonal, signaling, and downstream physiological responses. The magnitude and direction of these responses are context-dependent and may vary with plant species or genotype, microbial strain, drought intensity and duration, and developmental or stress stage. Abbreviations: ABA, abscisic acid; IAA, indole-3-acetic acid; CK, cytokinins; GA, gibberellins; JA, jasmonic acid; SA, salicylic acid; ACC, 1-aminocyclopropane-1-carboxylate; VOCs, volatile organic compounds; ROS, reactive oxygen species; NO, nitric oxide; MAPK, mitogen-activated protein kinase; DREB, dehydration-responsive element-binding protein; NAC, NAM/ATAF1/2/CUC2; WRKY, WRKY family transcription factors; MYB, MYB family transcription factors; bZIP, basic leucine zipper transcription factors; MYC2, basic helix–loop–helix transcription factor.

ACC deaminase-mediated regulation of ethylene is particularly relevant under drought conditions. By degrading ACC, the immediate precursor of ethylene, ACC deaminase-producing plant growth-promoting rhizobacteria can limit excessive stress-induced ethylene accumulation, thereby reducing ethylene-mediated inhibition of root elongation and premature senescence and helping maintain root development and physiological activity during prolonged water limitation [17,25]. Microbial communities can also influence strigolactone-mediated signaling, which contributes to root architecture, arbuscular mycorrhizal symbiosis, and rhizosphere communication. Modulation of strigolactone biosynthesis and perception may therefore affect root branching, fungal colonization, and microbial recruitment, linking hormonal regulation with microbiome assembly under drought stress [26]. Collectively, microbial biostimulants establish an integrated hormonal and signaling network that coordinates plant growth, water conservation, stress perception, and adaptive responses, thereby improving drought resilience under water-limited conditions. The complex hormonal and signaling networks regulated by microbial biostimulants during drought stress are summarized in Figure 2. These interconnected pathways coordinate plant growth, water conservation, stress signaling, and activation of drought-responsive genes, ultimately enhancing plant adaptation to water-deficit conditions.

### 2.3. Osmolyte Accumulation and Membrane Stability

Osmotic stress is one of the primary physiological consequences of drought, resulting from reduced soil water availability and the disruption of cellular water potential. As water availability declines, the delicate balance between cytosolic and vacuolar solutes is disturbed, leading to loss of cellular turgor, membrane destabilization, protein denaturation, oxidative damage, and impaired metabolic activity. To counteract these adverse effects, plants undergo osmotic adjustment by accumulating compatible solutes, commonly referred to as osmolytes, which enable cells to maintain water balance, preserve enzyme activity, and sustain essential physiological processes under dehydration stress [27]. Among the various compatible osmolytes, proline and glycine betaine are considered the principal metabolites that contribute to drought tolerance due to their multifunctional protective roles. These compounds lower cellular osmotic potential, facilitate water retention, stabilize proteins and nucleic acids, preserve membrane integrity, and protect the photosynthetic machinery from dehydration-induced injury. In addition, both osmolytes function as efficient scavengers of reactive oxygen species (ROS), thereby reducing oxidative damage and maintaining metabolic homeostasis during prolonged water deficit. Together with structural adaptations such as enhanced cuticular wax deposition, osmolyte accumulation substantially improves cellular hydration and minimizes electrolyte leakage under drought stress [28]. Besides proline and glycine betaine, polyamines, including putrescine, spermidine, and spermine, have emerged as important osmoprotective metabolites that participate in microbial-mediated drought adaptation. Polyamines contribute to membrane stabilization through electrostatic interactions with membrane phospholipids, proteins, and nucleic acids while simultaneously regulating ion homeostasis, antioxidant metabolism, and cellular redox balance. Increasing evidence indicates that beneficial microorganisms stimulate polyamine biosynthesis either directly through microbial metabolism or indirectly by modulating host plant metabolic pathways and phytohormone signaling. Elevated polyamine levels have been associated with enhanced osmotic adjustment, improved photosynthetic performance, reduced lipid peroxidation, and greater drought tolerance in numerous crop species [29].

Microbial effects on osmolyte accumulation and membrane stability are strongly context-dependent and should not be considered uniform across microbial groups or plant hosts. Studies involving bacterial inoculants such as *Bacillus*, *Paenibacillus*, and *Pseudomonas*, fungal biostimulants such as *Trichoderma*, and arbuscular mycorrhizal fungi (AMF) have reported changes in compatible-solute accumulation under drought, including increases in proline, glycine betaine, soluble sugars, trehalose, or polyamines; however, the specific metabolites affected and the magnitude of these responses vary among microbial taxa and strains, crop species and genotypes, drought intensity and duration, and experimental conditions [17,20,30]. Depending on the plant–microbe combination, these metabolic responses may contribute to osmotic adjustment and membrane stabilization and may occur together with changes in antioxidant enzyme activities, including superoxide dismutase (SOD), catalase (CAT), peroxidase (POD), and ascorbate peroxidase (APX). Microbial inoculation has also been associated with regulation of genes involved in osmolyte metabolism and with ABA-dependent and ABA-independent stress-signaling pathways, although the underlying regulatory responses differ among plant–microbe systems. Correspondingly, lower electrolyte leakage, higher relative water content (RWC), improved membrane stability, and enhanced recovery after rehydration have been reported in some inoculated plants relative to non-inoculated controls [31]; these outcomes should be interpreted in relation to the microbial strain, host genotype, severity and duration of water deficit, and whether the evidence was obtained under controlled-environment, greenhouse, or field conditions. Thus, microbial-mediated osmotic adjustment represents a context-dependent component of drought adaptation rather than a uniform response to microbial inoculation. Through these interconnected physiological and molecular mechanisms, microbial biostimulants can contribute to improved plant resilience and maintenance of growth under drought stress, although the magnitude and consistency of these benefits depend on the specific plant–microbe–environment combination.

### 2.4. Antioxidant Defense and Reactive Oxygen Species Scavenging

Drought stress disrupts cellular homeostasis by limiting carbon assimilation, impairing electron transport in chloroplasts and mitochondria, and altering multiple metabolic processes, consequently leading to the excessive production of reactive oxygen species (ROS). The major ROS generated under drought conditions include superoxide radicals (O_2_·^−^), hydrogen peroxide (H_2_O_2_), hydroxyl radicals (·OH), singlet oxygen (^1^O_2_), and alkoxy radicals, all of which can cause severe oxidative damage when produced in excess of the detoxification capacity of plant cells. Excessive accumulation of ROS induces lipid peroxidation, protein oxidation, nucleic acid damage, membrane destabilization, enzyme inactivation, and reduced photosynthetic efficiency, ultimately impairing plant growth and productivity [32]. Beyond their well-established cytotoxic effects, ROS also function as essential signaling molecules that regulate plant adaptation to drought stress. Controlled ROS production serves as a secondary messenger system that interacts with abscisic acid (ABA), calcium (Ca^2+^), nitric oxide (NO), mitogen-activated protein kinase (MAPK) cascades, and multiple transcription factor families, including WRKY, NAC, DREB, MYB, and bZIP proteins. These interconnected signaling pathways coordinate the expression of numerous drought-responsive genes involved in osmotic adjustment, antioxidant defense, hormonal regulation, membrane protection, and cellular homeostasis, thereby enabling plants to rapidly perceive and respond to environmental stress [33,34].

**Figure 3:**
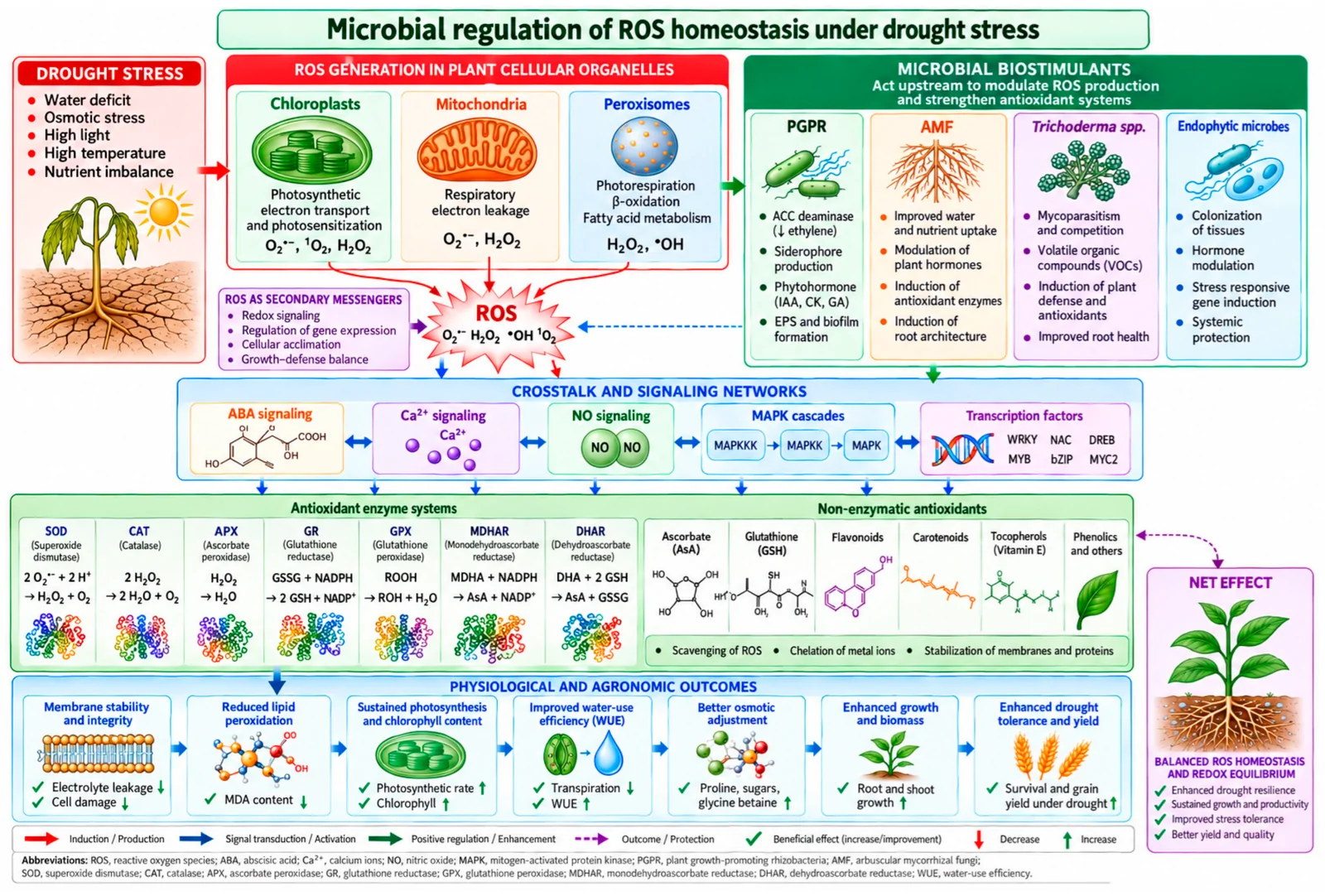
Microbial regulation of reactive oxygen species (ROS) homeostasis under drought stress. Drought stress stimulates excessive production of reactive oxygen species (ROS), including superoxide radicals (O_2_•^−^), hydrogen peroxide (H_2_O_2_), hydroxyl radicals (•OH), and singlet oxygen (^1^O_2_), primarily in chloroplasts, mitochondria, and peroxisomes. Besides causing oxidative damage, ROS also function as secondary messengers that activate complex stress-signaling networks involving abscisic acid (ABA), calcium (Ca^2+^), nitric oxide (NO), mitogen-activated protein kinase (MAPK) cascades, and drought-responsive transcription factors such as WRKY, NAC, DREB, MYB, bZIP, and MYC2. Beneficial microorganisms—including plant growth-promoting rhizobacteria (PGPR), arbuscular mycorrhizal fungi (AMF), *Trichoderma* spp., and endophytic microorganisms—modulate these signaling pathways by enhancing enzymatic antioxidants, including superoxide dismutase (SOD), catalase (CAT), ascorbate peroxidase (APX), glutathione reductase (GR), glutathione peroxidase (GPX), monodehydroascorbate reductase (MDHAR), and dehydroascorbate reductase (DHAR), together with non-enzymatic antioxidants such as ascorbate, glutathione, flavonoids, carotenoids, tocopherols, and phenolic compounds. These coordinated responses maintain cellular redox homeostasis, reduce lipid peroxidation and membrane damage, preserve photosynthetic performance, improve water-use efficiency and osmotic adjustment, and ultimately enhance plant growth, drought tolerance, and crop productivity under water-limited conditions. In the figure, red arrows indicate induction/production, blue arrows indicate signal transduction/activation, green arrows indicate positive regulation/enhancement, and purple dashed arrows indicate outcomes/protection. Check marks (✓) indicate beneficial or protective physiological effects associated with reduced oxidative damage and improved cellular integrity. Abbreviations: ROS, reactive oxygen species; ABA, abscisic acid; Ca^2+^, calcium ions; NO, nitric oxide; MAPK, mitogen-activated protein kinase; PGPR, plant growth-promoting rhizobacteria; AMF, arbuscular mycorrhizal fungi; SOD, superoxide dismutase; CAT, catalase; APX, ascorbate peroxidase; GR, glutathione reductase; GPX, glutathione peroxidase; MDHAR, monodehydroascorbate reductase; DHAR, dehydroascorbate reductase; WUE, water-use efficiency.

To maintain cellular redox homeostasis, plants have evolved a sophisticated antioxidant defense system consisting of both enzymatic and non-enzymatic antioxidants. The enzymatic antioxidant machinery primarily includes superoxide dismutase (SOD), catalase (CAT), ascorbate peroxidase (APX), glutathione reductase (GR), guaiacol peroxidase (GPX), monodehydroascorbate reductase (MDHAR), and dehydroascorbate reductase (DHAR), which function cooperatively through the ascorbate–glutathione cycle to detoxify ROS and protect cellular structures. In parallel, non-enzymatic antioxidants such as ascorbic acid, glutathione, cysteine, carotenoids, tocopherols, flavonoids, and phenolic compounds further contribute to ROS scavenging by maintaining intracellular redox balance and protecting biomolecules from oxidative injury [35]. Beneficial microorganisms play a central role in strengthening the antioxidant defense system of plants exposed to drought stress. Numerous microbial biostimulants, including plant growth-promoting rhizobacteria (PGPR), arbuscular mycorrhizal fungi (AMF), *Trichoderma* spp., and endophytic microorganisms, significantly enhance antioxidant enzyme activities while stimulating the accumulation of non-enzymatic antioxidants. These coordinated responses improve ROS detoxification, reduce lipid peroxidation, preserve membrane integrity, maintain chloroplast function, and sustain photosynthetic activity under water-deficit conditions [10,19]. Recent transcriptomic, proteomic, and metabolomic studies have demonstrated that microbial inoculation regulates the expression of numerous antioxidant-related genes and signaling pathways. Beneficial microorganisms activate ROS-scavenging enzymes through complex interactions involving ABA, jasmonic acid (JA), salicylic acid (SA), nitric oxide (NO), calcium-dependent protein kinases (CDPKs), MAPK cascades, and redox-sensitive transcription factors. This extensive signaling network coordinates antioxidant metabolism with osmotic adjustment, aquaporin regulation, hormonal crosstalk, and stress-responsive gene expression, thereby enhancing cellular protection against oxidative stress and improving drought adaptation [36]. In addition to enhancing antioxidant metabolism, microbial biostimulants alleviate oxidative stress indirectly by improving water-use efficiency, nutrient acquisition, photosynthetic performance, and mitochondrial function. These improvements reduce ROS overproduction at its source while maintaining cellular energy metabolism during prolonged drought. Consequently, inoculated plants generally exhibit lower concentrations of hydrogen peroxide (H_2_O_2_) and malondialdehyde (MDA), reduced electrolyte leakage, greater membrane stability, higher relative water content, and improved recovery following rehydration than non-inoculated plants [37]. Collectively, microbial-mediated regulation of ROS homeostasis represents one of the fundamental mechanisms underlying drought tolerance. Rather than merely scavenging excessive ROS, beneficial microorganisms establish a finely regulated redox network in which ROS function simultaneously as potentially harmful oxidants and indispensable signaling molecules. This balanced regulation enables plants to integrate antioxidant defense with hormonal signaling, osmotic adjustment, photosynthetic protection, and stress-responsive gene expression, thereby enhancing resilience, maintaining growth, and sustaining crop productivity under prolonged drought conditions [19,38]. The coordinated regulation of reactive oxygen species (ROS) homeostasis by microbial biostimulants under drought stress is summarized in Figure 3. The figure illustrates the integration of ROS production, antioxidant defense systems, stress-signaling pathways, and downstream physiological responses that collectively enhance drought tolerance.

### 2.5. Nutrient Acquisition and Nutrient Use Efficiency Under Drought

Microbial biostimulants substantially improve plant performance under drought stress by enhancing both nutrient acquisition and nutrient use efficiency (NUE). Water deficit not only limits soil water availability but also restricts nutrient diffusion, reduces mass flow, impairs root growth, and decreases the activity of nutrient transport systems, resulting in nutrient deficiencies that further aggravate drought-induced growth inhibition. Beneficial microorganisms alleviate these constraints by modifying rhizosphere processes, improving root architecture, and increasing nutrient bioavailability through multiple biochemical, physiological, and molecular mechanisms. Consequently, microbial inoculation enables plants to maintain nutrient homeostasis, sustain metabolic activity, and improve biomass production even under prolonged water limitation [17,22,39]. Plant growth-promoting rhizobacteria (PGPR), arbuscular mycorrhizal fungi (AMF), and endophytic microorganisms enhance nutrient acquisition through complementary mechanisms, including biological nitrogen fixation, phosphate solubilization, potassium mobilization, siderophore production, organic acid secretion, biological weathering of soil minerals, and mineralization of organic nutrients. These microbial processes increase the availability and uptake of essential macro- and micronutrients, including nitrogen (N), phosphorus (P), potassium (K), iron (Fe), zinc (Zn), manganese (Mn), and copper (Cu), which frequently become less available under drought conditions. Improved nutrient acquisition supports chlorophyll biosynthesis, photosynthetic efficiency, ATP production, enzyme activation, osmotic adjustment, antioxidant defense, and carbon metabolism, thereby enhancing overall plant drought tolerance and productivity [20].

Among microbial biostimulants, arbuscular mycorrhizal fungi (AMF), including species of *Rhizophagus*, *Funneliformis*, *Claroideoglomus*, *Gigaspora*, and *Acaulospora*, establish symbiotic associations with plant roots that can substantially improve nutrient and water acquisition. Their extensive extraradical hyphal networks explore soil volumes beyond root depletion zones, thereby enhancing the acquisition of relatively immobile nutrients, particularly phosphorus, zinc, and copper [40]. Simultaneously, AMF can improve soil–plant hydraulic conductance and root hydraulic properties, and provide additional pathways for water uptake and transport, thereby improving plant water status and drought tolerance [31]. Beneficial bacteria further enhance mycorrhizal performance through synergistic interactions within the rhizosphere. Several bacterial genera, including *Azospirillum*, *Pseudomonas*, *Burkholderia*, and *Bacillus*, function as mycorrhiza helper bacteria (MHB) by stimulating root branching, root hair development, fungal hyphal growth, and the production of signaling molecules and root exudates that facilitate AMF colonization. In return, AMF create favorable microhabitats that support bacterial proliferation and metabolic activity, establishing highly integrated fungal–bacterial networks that improve nutrient cycling, rhizosphere functionality, and plant drought resilience [41].

Beyond improving nutrient availability, recent transcriptomic and molecular studies have revealed that microbial biostimulants actively regulate the expression of numerous plant nutrient transporter genes. Beneficial microorganisms induce phosphate transporter genes (PHT1 family), nitrate transporter genes (NRT1 and NRT2 families), ammonium transporters (AMTs), potassium transporters (HAKs and AKTs), sulfate transporters (SULTRs), zinc-regulated transporter/iron-regulated transporter-like proteins (ZIPs), and iron transporters (IRT1), thereby increasing nutrient uptake efficiency under drought stress [23]. Simultaneously, microbial inoculation influences root nutrient sensing, hormone-dependent signaling pathways, and carbon allocation, allowing plants to optimize nutrient acquisition while minimizing energy expenditure under resource-limited conditions [42]. Microbial biostimulants also improve NUE by increasing nutrient assimilation, translocation, remobilization, and metabolic utilization rather than simply enhancing nutrient uptake. Improved NUE enables plants to produce greater biomass and maintain higher photosynthetic efficiency with lower nutrient inputs, thereby reducing dependence on chemical fertilizers and increasing agricultural sustainability [43]. These coordinated physiological and molecular responses integrate nutrient acquisition with water-use efficiency, antioxidant defense, osmotic adjustment, and hormonal regulation, thereby providing comprehensive protection against drought stress and maintaining crop productivity under water-limited environments. Overall, microbial biostimulants enhance drought tolerance through a highly integrated nutrient management strategy that combines improved nutrient availability, activation of nutrient transporter genes, enhanced nutrient uptake, optimized nutrient use efficiency, and coordinated fungal–bacterial interactions within the rhizosphere. These multifaceted mechanisms enable crops to maintain nutritional balance, metabolic activity, and sustainable productivity despite prolonged drought stress [44].

## 3. Plant–Microbiome Interactions Under Drought

Plant-associated microbial communities play a fundamental role in determining plant adaptation to drought by regulating rhizosphere processes, nutrient cycling, stress signaling, and microbial community dynamics. Rather than acting as isolated microorganisms, microbial biostimulants interact with the indigenous microbiome to reshape the composition, diversity, and functional capacity of microbial communities associated with plant roots. Increasing evidence indicates that drought resilience should be viewed as a characteristic of the plant holobiont, in which the host plant and its associated microbiome function as an integrated biological system that collectively responds to environmental stress [45]. Microbial inoculants can influence the assembly of plant-associated microbial communities by modifying the abundance and activity of resident microbial populations and, indirectly, root exudation patterns that affect microbial recruitment [26]. However, microbiome responses to drought are highly context-dependent and should not be interpreted as uniform stability or restructuring of a single “core microbiome.” The magnitude and direction of drought-induced changes can vary with crop species and genotype, plant developmental stage, drought intensity and duration, soil physicochemical properties, and the plant-associated compartment examined [46,47,48,49]. In particular, bulk soil, rhizosphere, rhizoplane (root surface), and root endosphere communities may exhibit distinct compositional and functional responses to water deficit because they differ in the strength of host selection and exposure to soil environmental conditions [48,49]. Reported patterns can also be influenced by methodological factors, including sampling strategy, sequencing platform, amplified marker region, taxonomic resolution, and bioinformatic processing, which should be considered when comparing microbiome responses among studies [49]. Thus, the apparent persistence of particular core or drought-associated taxa in some plant–microbiome systems should not be generalized across crops, compartments, or experimental conditions. Successful microbial inoculation consequently depends not only on the introduced strain but also on its compatibility and interactions with the indigenous microbiome within the relevant plant–soil compartment, together with plant genotype, soil properties, and environmental conditions [46,47]. These interacting factors can contribute to the variable performance of identical or similar microbial inoculants across soils, crops, and climatic conditions.

Arbuscular mycorrhizal fungi (AMF) further strengthen microbiome resilience through their hyphal networks that connect multiple plant root systems and create favorable microhabitats for diverse bacterial communities. These fungal networks facilitate nutrient redistribution, microbial dispersal, exchange of signaling molecules, and cooperative interactions among bacteria, fungi, and host plants, potentially resulting in highly interconnected microbial assemblages that may contribute to functional stability under drought stress. Such multispecies interactions may broaden the range of drought-protective functions through complementary effects on resource utilization, metabolic flexibility, and ecological stability within the rhizosphere [50]; however, direct comparisons indicate that multi-strain inoculants do not consistently outperform single strains, and any advantage is trait- and context-dependent (Section 3.2; Table 1). Recent advances in microbiome research have shifted attention from individual microbial inoculants toward microbiome engineering and the development of synthetic microbial communities (SynComs). These rationally designed microbial consortia combine microorganisms with complementary physiological traits, including nitrogen fixation, phosphate solubilization, phytohormone production, antioxidant stimulation, osmolyte biosynthesis, ACC deaminase activity, and pathogen suppression. SynComs have been proposed to offer greater functional stability, ecological resilience, and persistence than single-strain inoculants, making them promising tools for drought management [45]; however, these expected advantages have not yet been consistently demonstrated under field conditions and must be verified for each target crop and environment. Despite these advances, the effectiveness of microbial inoculants remains highly context-dependent because soil characteristics, climate, plant genotype, crop management practices, and the composition of indigenous microbial communities influence microbial establishment. Future research should therefore focus on understanding microbiome assembly rules, host–microbiome co-evolution, microbial network interactions, and precision microbiome engineering to develop robust microbial biostimulants that consistently enhance drought resilience under field conditions [51].

### 3.1. Root Exudation and Microbial Community Assembly

The quantity, composition, and temporal dynamics of root exudates are among the primary determinants governing the assembly, structure, and functional activity of rhizosphere microbial communities. Root exudates comprise a diverse array of low- and high-molecular-weight compounds, including sugars, amino acids, organic acids, phenolics, flavonoids, fatty acids, proteins, phytohormones, and secondary metabolites, which collectively serve as chemical signals mediating communication between plant roots and surrounding microorganisms. Consequently, changes in root exudation patterns strongly influence the selective recruitment of beneficial microorganisms and the establishment of plant-associated microbiomes [52]. Root exudation patterns differ considerably among plant species, genotypes, developmental stages, and environmental conditions, resulting in highly dynamic microbial communities throughout the plant life cycle. Under drought stress, both the quantity and chemical composition of root exudates are substantially modified, with increased secretion of compatible solutes, sugars, amino acids, organic acids, phenolic compounds, flavonoids, and specialized secondary metabolites. These compounds function as selective chemical signals and nutritional resources that favor the recruitment and establishment of drought-adapted beneficial microorganisms, thereby reshaping rhizosphere community composition and enhancing rhizosphere functionality [51]. These changes support microbial survival under water-deficit conditions while simultaneously enhancing nutrient cycling, antioxidant metabolism, osmotic adjustment, and stress-responsive signaling within the rhizosphere.

Recent studies suggest that plants actively manipulate their rhizosphere microbiome through a process often described as the “cry for help” hypothesis, whereby drought-stressed plants alter their root exudation profiles to recruit microorganisms that alleviate environmental stress. Beneficial microorganisms subsequently enhance plant adaptation by improving nutrient acquisition, phytohormone production, antioxidant defense, osmotic adjustment, and disease suppression, establishing a positive feedback loop that further strengthens plant resilience. Rather than being passive responses, these dynamic plant–microbe interactions represent an adaptive strategy that enables the plant holobiont to respond rapidly to changing environmental conditions [53]. Although numerous studies have investigated the relationship between root exudate composition and rhizosphere microbial diversity, most have evaluated microbial communities at a single sampling point. Consequently, the temporal dynamics of root exudation and microbiome assembly throughout plant development remain insufficiently understood. Growing evidence indicates that microbial recruitment is a continuous process, with plants continually modifying exudation patterns in response to developmental stage, physiological status, and environmental cues. These temporal changes progressively reshape microbial community composition and function, enabling plants to recruit microorganisms optimally adapted to current environmental conditions [54].

**Figure 4:**
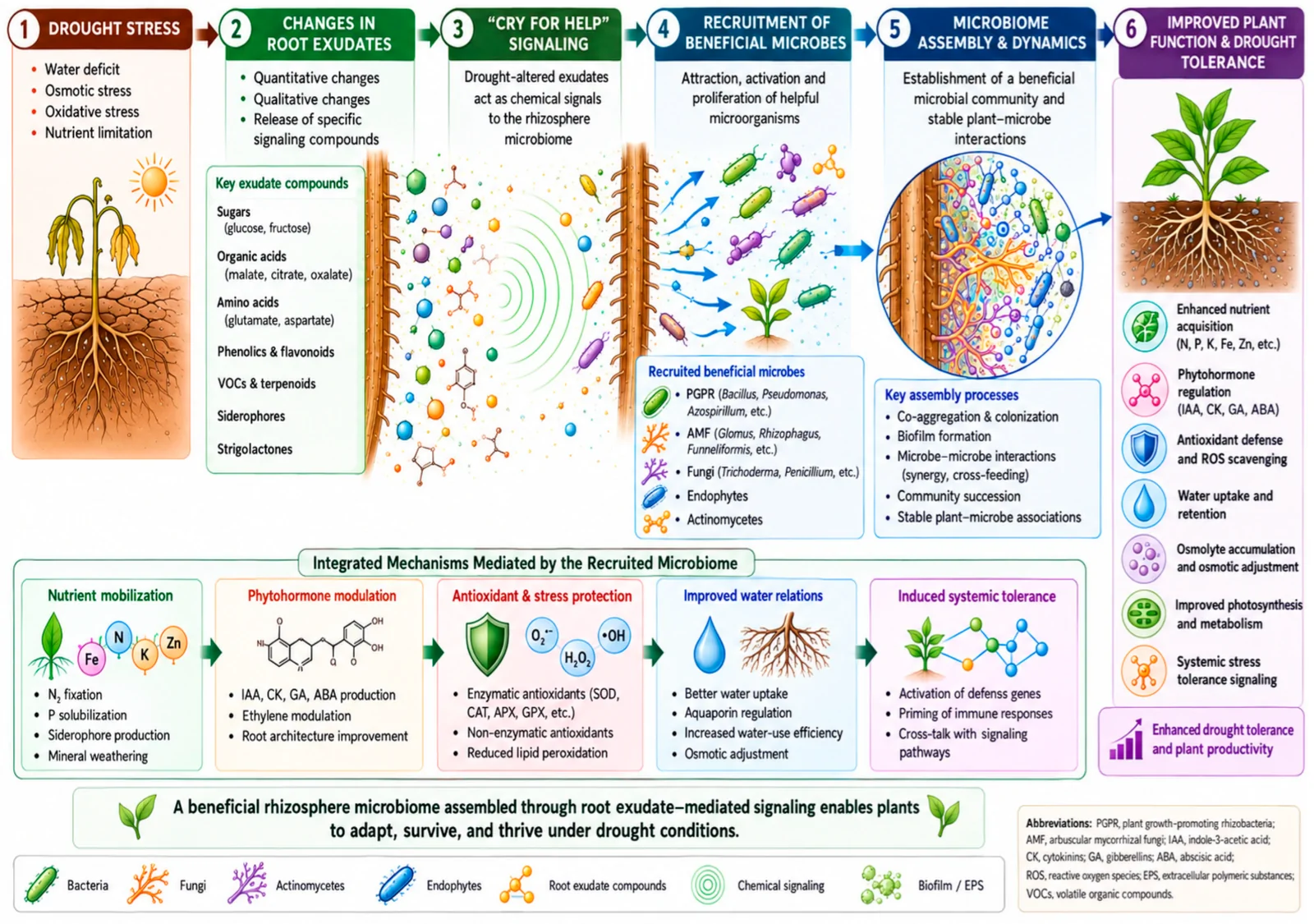
Root exudate-mediated recruitment and assembly of beneficial rhizosphere microbiomes under drought stress. Drought stress induces quantitative and qualitative changes in root exudate composition, including the release of sugars, organic acids, amino acids, phenolic compounds, flavonoids, volatile organic compounds (VOCs), siderophores, and signaling molecules that function as a plant’s “cry for help.” These drought-induced exudates selectively recruit beneficial microorganisms, including plant growth-promoting rhizobacteria (PGPR), arbuscular mycorrhizal fungi (AMF), *Trichoderma* spp., endophytes, and actinomycetes, thereby establishing a beneficial rhizosphere microbiome. Subsequent microbial colonization, biofilm formation, and interspecies interactions promote nutrient mobilization, phytohormone modulation, antioxidant defense, improved water relations, osmotic adjustment, and activation of systemic stress responses. Collectively, these coordinated microbiome-mediated processes enhance root function, photosynthetic performance, water-use efficiency, drought tolerance, and crop productivity under water-limited environments. Arrows indicate the sequential progression and directional relationships among drought stress, changes in root exudation, chemical signaling, microbial recruitment and assembly, microbiome-mediated mechanisms, and resulting plant responses. Microbial, molecular, root, nutrient, water, shield, and related graphical symbols represent the corresponding biological components and processes indicated by their adjacent labels; the concentric-circle symbol represents chemical signaling, and the biofilm/EPS symbol represents microbial aggregation and extracellular polymeric substances. Abbreviations: PGPR, plant growth-promoting rhizobacteria; AMF, arbuscular mycorrhizal fungi; VOCs, volatile organic compounds; ROS, reactive oxygen species; IAA, indole-3-acetic acid; CK, cytokinins; GA, gibberellins; ABA, abscisic acid; EPS, extracellular polymeric substances.

Despite growing understanding of root exudate-mediated microbial recruitment, the influence of multi-species microbial consortia on root exudation dynamics and downstream microbiome assembly remains largely unexplored. Future studies integrating metabolomics [55], microbiome sequencing, transcriptomics, stable isotope labeling, and network analysis will provide deeper insights into the mechanisms governing rhizosphere assembly under drought stress. Such multidisciplinary approaches will facilitate the rational design of next-generation microbial biostimulants and synthetic microbial communities that consistently improve drought resilience across diverse agricultural systems [51]. The sequential processes through which drought-induced changes in root exudation recruit beneficial microorganisms, shape rhizosphere microbiome assembly, and enhance plant drought tolerance are summarized in Figure 4. The figure highlights the central role of root exudates as chemical signals that link plant stress perception to microbiome-mediated adaptive responses.

### 3.2. Microbial Networking and Synergistic Consortia

Drought stress profoundly alters the composition, diversity, and functional organization of root-associated microbial communities, thereby changing microbial interactions that influence plant adaptation to water-deficit conditions. Reduced soil moisture limits microbial mobility, nutrient diffusion, and metabolic activity while increasing spatial heterogeneity within the rhizosphere. Despite these environmental constraints, interactions among beneficial microorganisms can generate complementary or synergistic functions that contribute to plant adaptation under water deficit. Microbial consortia may combine traits such as biological nitrogen fixation, phosphate solubilization, phytohormone modulation, ACC deaminase activity, osmolyte regulation, antioxidant stimulation, and pathogen suppression. Such functional complementarity provides a mechanistic basis for expecting multi-strain inoculants to produce broader or more stable responses than individual strains. However, experimental comparisons indicate that consortium superiority is not universal and depends on the compatibility and functional complementarity of the component microorganisms, host plant, drought regime, soil and nutrient conditions, and response variable evaluated [56,57,58,59]. In some plant–microbe systems, consortia have produced greater improvements in biomass, photosynthetic performance, antioxidant activity, root development, or survival than their constituent single strains, whereas in others the advantage has been limited to particular traits or environmental conditions and has not translated into increased yield [56,57,58,59]. Representative direct comparisons between microbial consortia and single-strain inoculants under water-deficit conditions are summarized in Table 1.

**Table 1:** Comparative performance of microbial consortia and single-strain inoculants in plants exposed to drought stress.

Inoculant Type/Comparison	Crop	Drought Conditions/Experimental System	Response Variables	Comparative Outcome/Effect Size	Ref.
Four-species bacterial consortium SPMX (*Stenotrophomonas rhizophila*, *Xanthomonas retroflexus*, *Microbacterium oxydans*, and *Paenibacillus amylolyticus*) vs. individual component strains	*Arabidopsis thaliana* Col-0	Pot experiment; water withheld for 21 days followed by 7 days of rehydration	Survival, fresh weight, rosette diameter, chlorophyll, ABA signaling	Consortium-treated plants showed 71.6 ± 6.3% survival after drought and rehydration compared with 35.8 ± 6.6% in non-inoculated controls; individual strains did not significantly improve survival. Under drought, consortium treatment increased fresh weight by approximately 2-fold and rosette diameter by approximately 1.5-fold relative to controls.	[56]
Individual PGPR strains (*Bacillus* sp. MN-54, *Enterobacter* sp., *Pseudomonas fluorescens* 1) vs. two- and three-strain bacterial consortia	Maize (*Zea mays* L.); drought-tolerant NK-6654 and drought-sensitive SD-626 hybrids	Pot experiment under water-deficit conditions; bacterial strains pre-screened at 0–30% PEG-6000	Biomass, RWC, photosynthetic pigments, photosynthetic activity, CAT, SOD, GPX	Individual strains increased photosynthetic activity by 25–39% and antioxidant activity by 28–38%, whereas consortia produced increases of 47–61% and 42–62%, respectively. The MN-54 + *Enterobacter* consortium increased RWC by up to 35%, shoot length by 44%, root length by 52%, shoot dry weight by 44%, and root dry weight by 38% relative to the uninoculated drought control.	[57]
Six individual bacterial isolates vs. three beneficial microbial consortia (BMc1, BMc2, BMc3); BMc1 and BMc2 were three-bacterial-member consortia, whereas BMc3 comprised *Bacillus atrophaeus*, *Pseudomonas* sp., and *Trichoderma harzianum*	Maize (*Zea mays* L.) B73	Greenhouse; loam and sandy substrates; drought imposed by withholding irrigation during the final week of a 5-week experiment	Shoot fresh/dry mass, root growth, rhizosphere colonization, microbial community composition	Single bacterial isolates did not significantly improve maize growth in the corresponding single-inoculation experiments. Under drought, BMc1 increased shoot dry mass by 41% in loam relative to the non-inoculated control. Consortium effects were substrate-dependent, with no significant growth-promoting effect detected in sand.	[58]
Six individual bacterial/fungal inoculants vs. ten microbial consortia, including bacterial–bacterial and bacterial/fungal/AMF combinations	Potato (*Solanum tuberosum* L.)	Controlled experiment: 70% reduction in water supply for 6 weeks; additional field evaluation; effects also evaluated under different N forms	Shoot/root growth, P and K nutrition, leaf drought damage, recovery, tuber biomass/yield	P nutritional status was improved above the deficiency threshold by 3 of 6 single inoculants vs. 8 of 10 consortia. Consortia KH and P8R reduced drought-induced leaf damage by 41% and 37%, respectively, while the single strain FZB42 reduced irreversible damage after recovery by 40% and consortium P&R by 50%. However, neither single nor consortium inoculation mitigated the drought-induced reduction in tuber yield, demonstrating that consortium advantages were trait- and context-dependent.	[59]

Collectively, these studies indicate that functional complementarity among consortium members can enhance drought-protective responses, but consortium superiority should not be regarded as universal. Benefits may be restricted to particular physiological traits, host–microbe combinations, or environmental conditions and do not necessarily translate into improved yield under drought [56,57,58,59].

Recent advances in microbial ecology indicate that microbial communities exhibit remarkable functional redundancy, in which multiple taxa perform similar ecological functions. This redundancy enhances ecosystem resilience because other microbial species can compensate for the loss or reduced activity of one species within the consortium. Consequently, microbial networks can maintain relatively stable nutrient cycling, hormonal regulation, and stress-responsive functions despite environmental disturbances such as prolonged drought. In addition, cooperative metabolic interactions, including cross-feeding, metabolite exchange, quorum sensing, and biofilm formation, may further strengthen community stability and improve the persistence of beneficial microorganisms within the rhizosphere [60,61]. An emerging strategy for improving drought tolerance involves developing SynComs that combine microorganisms with complementary physiological traits. Rationally designed SynComs may include microorganisms capable of biological nitrogen fixation, phosphate solubilization, siderophore production, phytohormone biosynthesis, ACC deaminase activity, exopolysaccharide production, antioxidant stimulation, osmolyte accumulation, and disease suppression. Compared with single-strain inoculants, appropriately designed SynComs may provide broader functional diversity and greater functional redundancy; however, these properties do not necessarily result in superior plant performance. Their effectiveness depends on compatibility among consortium members, successful establishment within the resident microbiome, host genotype, soil and nutrient conditions, drought regime, and the response variable evaluated. Direct comparative studies therefore remain necessary to determine when functional complementarity within a consortium translates into a measurable advantage over its constituent strains (Table 1). Microbial networking also contributes to enhanced resilience by dynamically regulating microbial activity across repeated drought cycles. Cooperative microbial communities can shift between metabolically active and dormant states in response to fluctuations in soil moisture while maintaining overall community functionality. Such dynamic regulation may allow microbial consortia to rapidly reactivate following rehydration, preserving beneficial plant–microbe interactions and facilitating long-term adaptation to recurrent drought events. These adaptive characteristics highlight the importance of microbial ecological interactions in sustaining plant productivity under increasingly variable climatic conditions.

In addition to their potential effects on drought tolerance, integrated microbial inoculants may contribute to broader ecosystem functions in agricultural soils. Beneficial microbial consortia can stimulate nutrient mineralization, enhance soil aggregation through exopolysaccharide production, improve carbon sequestration, increase soil organic matter turnover, promote hydrocarbon degradation, and facilitate nutrient transfer within complex microbial communities inhabiting non-sterile soils. Consequently, microbial networking may contribute not only to enhanced plant performance but also to improved soil health, ecosystem multifunctionality, and long-term agricultural sustainability [62]. Overall, exploiting microbial networking and synergistic interactions represents a promising strategy for developing next-generation microbial biostimulants. Future inoculant design should consider both well-characterized single strains and functionally compatible microbial consortia, and the choice between them should be based on direct comparative evidence for the target crop, soil, and drought regime rather than on an assumed general superiority of multi-strain products. Such approaches may contribute to sustainable crop production systems that can withstand increasingly frequent and severe drought events.

### 3.3. Context-Dependence: Plant Genotype, Soil Type, and Water Regime

The complex interactions among plant genotype, soil characteristics, environmental conditions, and the indigenous microbiome strongly influence the effectiveness of microbial biostimulants in enhancing drought tolerance. Although numerous beneficial microorganisms have demonstrated considerable potential under controlled experimental conditions, their performance under field environments frequently varies because plant–microbe interactions are highly context-dependent. Consequently, understanding the interplay between host genetics, soil properties, microbial community composition, and water availability is essential for the successful application of microbial biostimulants in sustainable agriculture [63]. Plant genotype represents one of the principal factors governing microbiome assembly and microbial colonization. Different plant species and cultivars release distinct root exudate profiles that selectively recruit specific microbial taxa, resulting in genotype-dependent microbial communities with unique functional capacities. Modern crop breeding and domestication have also altered the diversity and composition of plant-associated microbiomes, often reducing the recruitment of beneficial microorganisms compared with their wild relatives. Consequently, the responses of microbial inoculants may differ substantially among cultivars due to variation in root architecture, nutrient requirements, hormone signaling, immune responses, and drought adaptation strategies [64].

Soil physicochemical properties further determine the establishment and persistence of microbial inoculants. Soil texture, pH, organic matter content, nutrient availability, salinity, temperature, aeration, and native microbial diversity all influence microbial survival, colonization efficiency, and ecological competitiveness. Beneficial microorganisms introduced into agricultural soils must compete with well-established indigenous microbial communities, and their persistence often depends on compatibility with resident microbiota and local environmental conditions. These interactions largely explain why microbial inoculants that perform well in one soil type may be less effective in another [65]. Water regime is another critical determinant of microbial performance. The intensity, duration, frequency, and timing of drought events influence both plant physiology and microbial community dynamics, thereby modifying the effectiveness of microbial biostimulants. Mild or transient drought conditions may stimulate beneficial plant–microbe interactions, whereas prolonged or severe drought can reduce microbial activity, alter community composition, and limit microbial colonization. Conversely, microbial communities adapted to arid environments often exhibit greater persistence and functional stability during repeated drought cycles, highlighting the importance of selecting native or locally adapted microbial strains for field applications [66]. The interaction between beneficial microorganisms and host plants extends beyond nutrient acquisition and nitrogen fixation. For example, both *Azospirillum* spp. and *Rhizobium* spp. have been shown to improve the growth and drought tolerance of water-stressed tomato (*Solanum lycopersicum*) independently of their nitrogen-fixing capacity, indicating that phytohormone production, ACC deaminase activity, antioxidant stimulation, osmotic adjustment, and modulation of stress-responsive signaling pathways contribute substantially to plant adaptation under drought conditions [44,67]. Similar mechanisms have been reported for arbuscular mycorrhizal fungi, *Trichoderma* spp., and endophytic microorganisms, demonstrating that multiple microbial groups employ complementary physiological strategies to improve drought resilience.

Recent advances in plant–microbiome research indicate that microbial inoculants should no longer be evaluated in isolation from their environmental context. Instead, successful implementation requires consideration of the integrated interaction among plant genotype, soil properties, indigenous microbiomes, management practices, and climatic conditions, commonly described as the G × S × M × E (Genotype × Soil × Microbiome × Environment) framework. This systems-based perspective provides a more realistic approach to predicting microbial performance under field conditions and offers opportunities to develop precision microbial biostimulants tailored to specific agroecosystems. To operationalize the G × S × M × E framework, inoculant selection should be based on measurable descriptors within each component rather than on categorical matching alone. For genotype (G), relevant variables include crop species and cultivar, root-system architecture, phenology, drought sensitivity, root-exudate characteristics, and reproducible physiological responses to inoculation [63,64]. Soil (S) characterization should include pH, texture, organic matter, electrical conductivity, nutrient availability, water-holding capacity, and baseline soil moisture, because these properties influence microbial survival, colonization, and functional performance [63,65]. Microbiome (M) assessment should consider the composition and diversity of indigenous rhizosphere and root-associated communities, abundance of functionally relevant taxa or genes, compatibility of the introduced strain with resident microorganisms, and post-inoculation establishment or persistence [45,47,51]. Environmental (E) descriptors should include drought intensity, duration, frequency and timing, soil water potential or volumetric water content, temperature, precipitation, evaporative demand, and irrigation regime [63,66]. These measurable variables, together with corresponding analytical approaches and proposed decision criteria for inoculant selection, are summarized in Table 2. These variables can be incorporated into factorial experiments and multivariate or predictive models to identify G × S × M × E combinations associated with reproducible inoculant performance [68,69,70]. Rather than applying universal numerical thresholds across crops and environments, decision criteria should be established for each target production system using predefined endpoints and appropriate uninoculated and, where relevant, well-watered controls. Candidate inoculants should advance from controlled screening to multi-location field validation when they demonstrate reproducible establishment together with statistically supported improvements in predefined drought-response endpoints, such as biomass or yield stability, relative water status, water-use efficiency when directly measured, or recovery after rehydration [69,70]. Accordingly, the operational criteria proposed in Table 2 are intended as a flexible decision-support framework rather than universal numerical thresholds. This approach enables microbial inoculants to be matched with defined crop–soil–microbiome–environment combinations while retaining the context dependence inherent in plant–microbe interactions.

**Figure 5:**
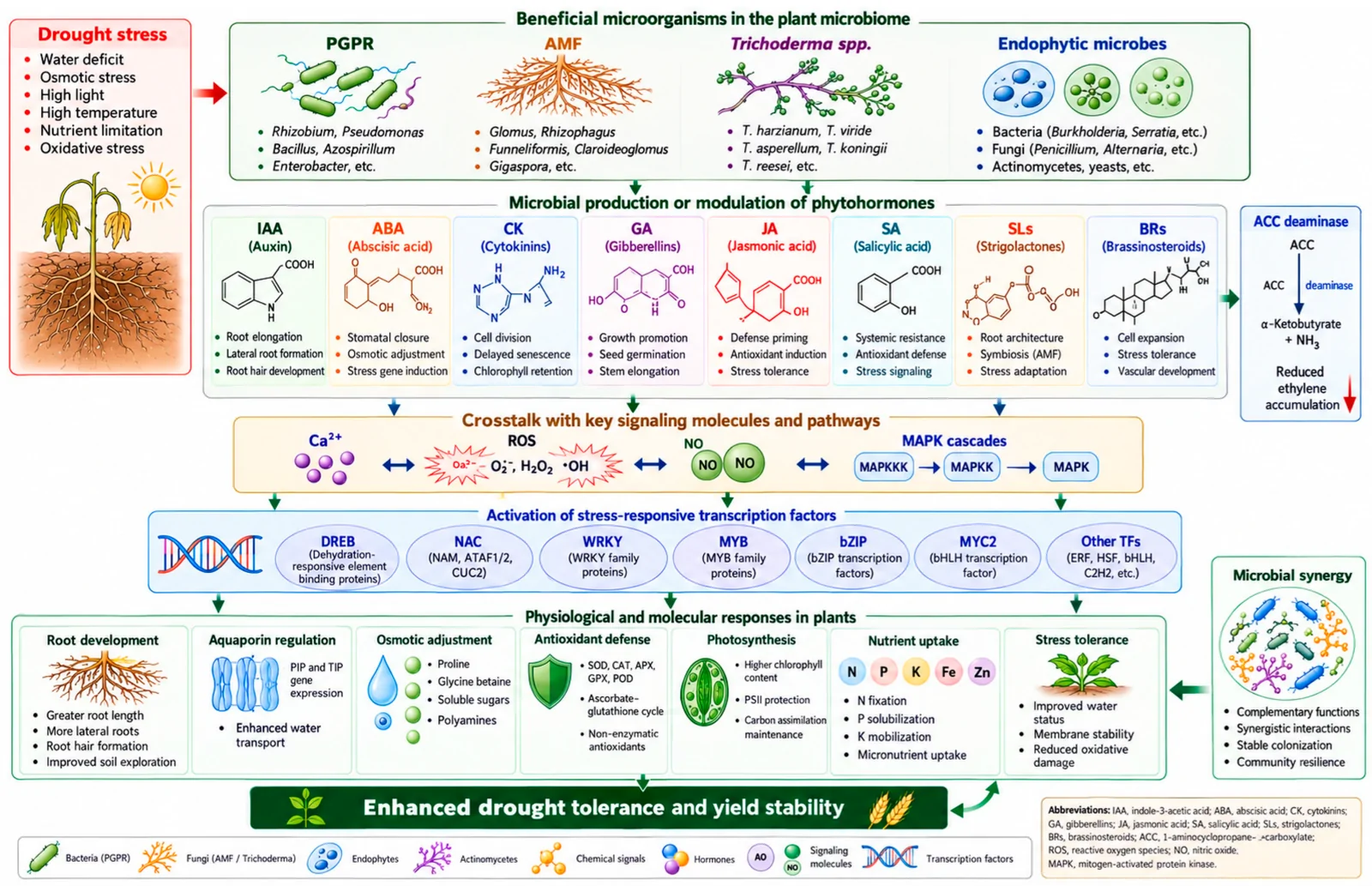
Determinants of microbial biostimulant performance under drought stress: A Genotype × Soil × Microbiome × Environment (G × S × M × E) framework. Dynamic interactions among plant genotype, soil properties, microbiome composition, environmental conditions, and agricultural management practices govern the performance of microbial biostimulants. Plant genotype influences root architecture, root exudation patterns, immune responses, and microbial recruitment. Soil physicochemical characteristics regulate microbial survival, colonization, nutrient availability, and water retention capacity, while environmental factors—including drought intensity, temperature, light, and precipitation—shape microbial activity and plant stress responses. Interactions between indigenous microbial communities and introduced microbial inoculants determine microbiome establishment, functional stability, and ecosystem resilience. Agronomic practices such as fertilization, irrigation, tillage, crop rotation, pesticide use, and formulation strategy further influence these interactions. Together, these factors regulate microbial functions in the rhizosphere, including nutrient solubilization, phytohormone production, ACC deaminase activity, biofilm formation, siderophore production, antioxidant defense, and stress signaling. The integration of these processes ultimately enhances microbial establishment, nutrient acquisition, hormonal regulation, antioxidant capacity, plant physiological adaptation, drought resilience, yield stability, and sustainable crop production under water-limited environments. Operationalization of this framework requires quantitative characterization of genotype-, soil-, microbiome-, and environment-related variables and validation of candidate inoculants against predefined, system-specific performance criteria, as summarized in Table 2. Abbreviations: G, plant genotype; S, soil properties; M, microbiome; E, environment; PGPR, plant growth-promoting rhizobacteria; AMF, arbuscular mycorrhizal fungi; ACC, 1-aminocyclopropane-1-carboxylate; EPS, extracellular polymeric substances; ROS, reactive oxygen species; IAA, indole-3-acetic acid; CK, cytokinins; GA, gibberellins; ABA, abscisic acid; JA, jasmonic acid; SA, salicylic acid; VOCs, volatile organic compounds.

Although numerous studies have demonstrated the effectiveness of microbial biostimulants under greenhouse and laboratory conditions, their successful translation into agricultural practice requires extensive multi-location field validation across diverse soil types, climatic regions, irrigation regimes, and crop genotypes, which is not always achieved. Future research should therefore integrate field experimentation with multi-omics approaches, ecological modeling, and precision agriculture technologies to identify microbial inoculants that provide consistent, reproducible drought tolerance under variable environmental conditions [69]. Such integrative approaches will facilitate the development of next-generation microbial biostimulants with greater reliability, scalability, and practical applicability for climate-resilient agriculture [70]. The effectiveness of microbial biostimulants under drought stress is determined by complex interactions among plant genotype, soil characteristics, indigenous and introduced microbiomes, environmental conditions, and agronomic management practices. These multidimensional interactions are summarized in Figure 5, which presents a systems-level framework illustrating how biological and environmental factors collectively regulate microbial establishment, plant physiological responses, and drought resilience.

## 4. Sustainable Agricultural Applications

The successful agricultural application of microbial biostimulants remains strongly influenced by field and environmental conditions. Large-scale field experiments demonstrate that treatment efficacy can vary among locations, with responses ranging from highly beneficial to neutral or inconsistent depending on plant genotype, soil physicochemical properties, indigenous microbial communities, climatic conditions, irrigation regime, and agricultural management practices [71,72]. Consequently, greater emphasis should be placed on conducting long-term, multi-location field trials to identify the key drivers governing microbial establishment, persistence, and functional performance under diverse agroecosystems. Compatibility with existing crop management practices is equally important for successful field implementation. Microbial biostimulants should be evaluated not only as standalone products but also in combination with irrigation management, reduced fertilizer inputs, organic amendments, conservation agriculture, and integrated pest management (IPM) programs. Such integrated management strategies frequently provide greater agronomic and economic benefits than individual interventions by simultaneously improving soil fertility, water-use efficiency, nutrient availability, and crop resilience to multiple environmental stresses.

The regulatory landscape also plays a decisive role in the commercialization and adoption of microbial biostimulants. Product registration, quality control, microbial identification, biosafety evaluation, manufacturing consistency, and international regulatory harmonization remain major challenges for the microbial biostimulant industry. The implementation of regulatory frameworks such as the European Union Fertilizing Products Regulation (EU) 2019/1009 has provided standardized definitions and quality requirements for microbial biostimulants, facilitating product commercialization while ensuring efficacy, safety, and environmental sustainability. Continued harmonization of international regulations will be essential for expanding global markets and increasing farmer confidence in microbial technologies. Advances in precision agriculture, microbiome engineering, SynComs, artificial intelligence-assisted microbial selection [73], and multi-omics technologies [74] are expected to accelerate the development of next-generation microbial biostimulants. These innovations will enable the design of highly specialized microbial formulations tailored to specific crop genotypes, soil characteristics, climatic conditions, and management systems, thereby improving field consistency and maximizing drought resilience under future climate scenarios. Integrating microbial biostimulants into climate-smart agricultural practices will therefore represent an important strategy for achieving sustainable crop production while reducing environmental impacts and increasing global food security.

### 4.1. Field Performance and Yield Stability

Field evaluations of microbial biostimulants under water-limited conditions have been conducted across different crops, microbial groups, geographical locations, and drought or irrigation regimes, although the magnitude and statistical significance of yield responses vary considerably among studies [75,76,77,78]. Representative field trials are summarized in Table 3, including crop, inoculant, experimental location, drought or irrigation treatment, yield response, and statistical outcome. Direct field evidence demonstrates that microbial inoculation can significantly improve yield under particular crop–microbe–environment combinations, whereas other experiments have reported non-significant or management-dependent responses, emphasizing that field efficacy should not be generalized across agroecosystems [6,79,80,81,82,83,84]. Accordingly, field performance should be interpreted in relation to the microbial inoculant, crop genotype, drought regime, soil and climatic conditions, and agronomic management rather than solely on the basis of results obtained under controlled conditions.

As summarized in Table 3, field responses to microbial biostimulants range from significant improvements in crop yield under particular water-deficit conditions to non-significant or management-dependent effects [6,79,80,81,82,83,84]. For example, bacterial inoculation increased wheat grain yield most strongly under moderate drought but did not significantly improve yield under severe drought [79], while the performance of a synthetic microbial consortium in wheat differed among agroecological, conventional, and no-till management systems [83]. Similarly, multi-location soybean trials demonstrated positive yield responses to Bacillus-based inoculants, but the magnitude of the response varied among locations and growing seasons [84]. These findings reinforce that microbial biostimulant efficacy under field conditions is determined by interactions among drought severity, crop genotype, soil and climatic conditions, microbial composition, and agronomic management rather than by inoculation alone.

**Figure 6:**
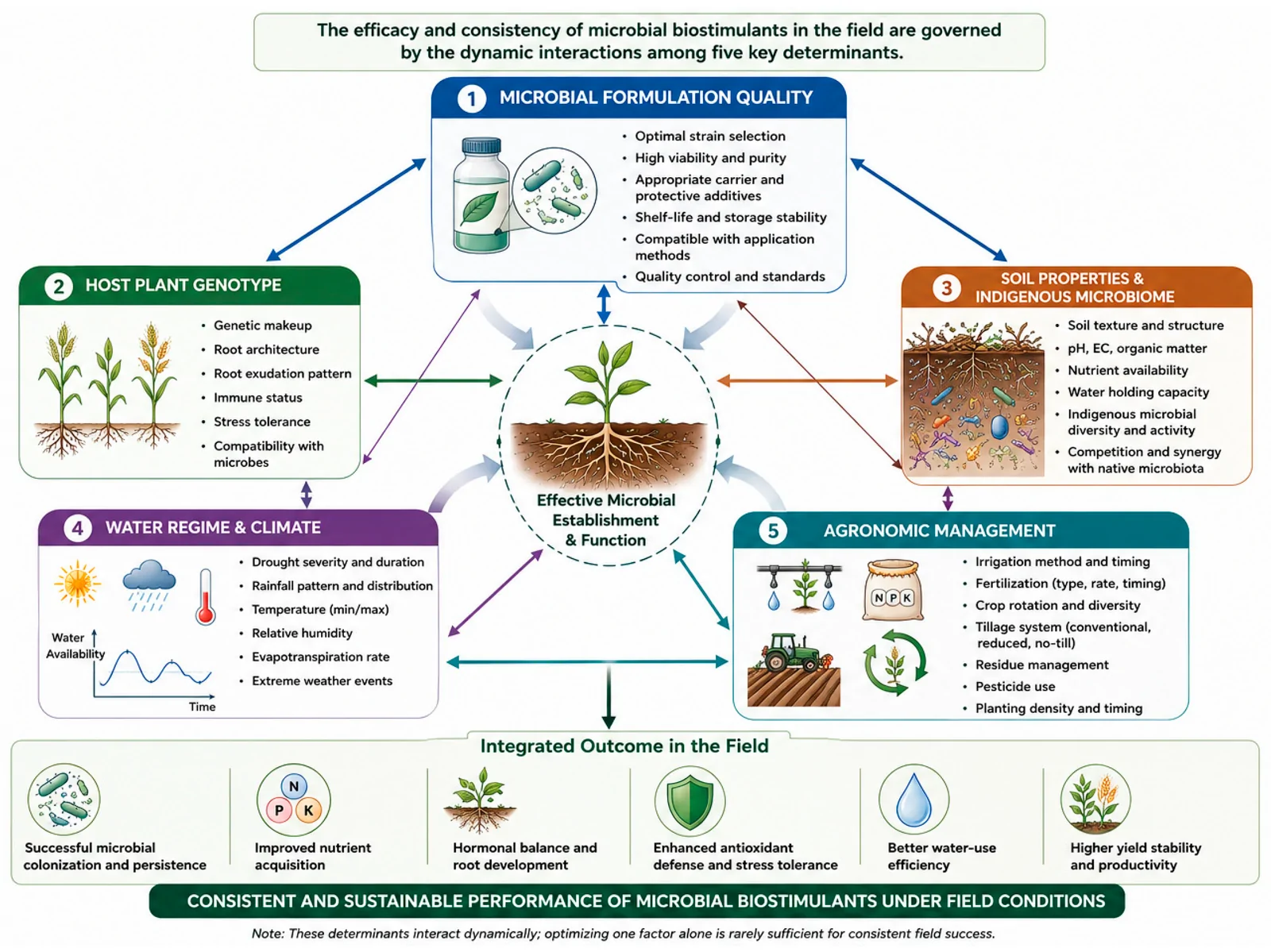
Five major determinants of successful field performance of microbial biostimulants under drought conditions. The consistency of microbial biostimulant performance under field conditions depends on dynamic interactions among five major determinants: (1) microbial formulation quality, including strain selection, microbial viability, carrier composition, shelf life, and product quality; (2) host plant genotype, which influences root architecture, root exudation patterns, immune responses, and compatibility with beneficial microorganisms; (3) soil physicochemical properties and indigenous microbiomes that regulate microbial survival, colonization, nutrient availability, and ecological interactions; (4) water regime and climatic conditions, including drought severity, temperature, rainfall distribution, and environmental variability; and (5) agronomic management practices such as irrigation, fertilization, crop rotation, tillage, residue management, and pesticide use. The integration of these factors determines microbial establishment, nutrient acquisition, hormonal regulation, antioxidant defense, water-use efficiency, drought resilience, yield stability, and sustainable crop productivity under water-limited agricultural systems. Abbreviations: EC, electrical conductivity; PGPR, plant growth-promoting rhizobacteria; NPK, nitrogen–phosphorus–potassium fertilizer.

Increasing evidence indicates that yield stability, rather than maximum yield potential alone, represents one of the greatest advantages of microbial biostimulants under drought stress. Such stability becomes increasingly important amid climate change, where greater variability in rainfall distribution and more frequent droughts threaten agricultural production systems. Despite encouraging results from field studies, considerable variation in microbial biostimulant performance persists across locations, seasons, soil types, crop genotypes, and irrigation regimes. This variability reflects the highly context-dependent nature of plant–microbe interactions and highlights the importance of considering local environmental conditions when selecting microbial inoculants [78]. Future research should therefore prioritize long-term, multi-environment field experiments integrating plant genotype, indigenous microbiome composition, soil physicochemical properties, climatic variables, and management practices to identify the principal drivers of field consistency and microbial persistence. The anticipated increase in drought frequency due to global climate change further underscores the importance of microbial biostimulants as components of climate-smart agriculture. While substantial efforts continue to focus on developing drought-tolerant cultivars through conventional breeding and molecular approaches, microbial biostimulants provide a complementary strategy by enhancing the adaptive capacity of existing crop varieties through beneficial plant–microbe interactions. Their integration with precision irrigation, conservation agriculture, optimized fertilization [85], and integrated pest management (IPM) [86] has considerable potential to improve agricultural resilience while reducing environmental impacts. Consequently, microbial biostimulants should increasingly be viewed as components of integrated crop management systems rather than standalone agricultural inputs [30]. Looking forward, advances in precision agriculture, remote sensing, soil moisture monitoring, artificial intelligence (AI)-assisted decision support, and microbiome-informed crop management are expected to improve the precision and consistency of microbial biostimulant applications under field conditions. Integrating microbial technologies with digital agriculture could enable more informed selection of microbial formulations, application timing, irrigation scheduling, and site-specific crop management, thereby enhancing drought resilience, yield stability, and long-term sustainability under increasingly variable climatic conditions [87]. The successful field performance of microbial biostimulants depends on the interaction of multiple biological, environmental, and management factors rather than on microbial inoculation alone [88]. The principal determinants governing microbial establishment, persistence, and agronomic effectiveness under drought conditions are summarized in Figure 6.

### 4.2. Formulation Types, Delivery Methods, and Applicability

The field efficacy and commercialization of microbial biostimulants depend strongly on formulation technologies that maintain microbial viability and functional activity during storage, transportation, and application. Effective formulations should protect microorganisms from environmental stress and facilitate their establishment and persistence after application, thereby improving the consistency of microbial performance under field conditions. Microbial biostimulants can be delivered through liquid suspensions, wettable powders, granules, peat- or biochar-based carriers, seed coatings, alginate beads, polymer encapsulation systems, and freeze-dried formulations. The choice of formulation influences microbial viability, shelf life, application efficiency, and establishment under field conditions. Quantitative formulation requirements vary substantially with microbial species and strain, carrier composition, production process, packaging, intended application, and storage conditions. Consequently, no single set of numerical specifications can be applied universally to all microbial biostimulants. Nevertheless, several parameters provide useful benchmarks for formulation development and quality assessment. Shelf-life targets reported for microbial inoculants range from approximately 2–3 months at ambient temperature to 1–2 years or longer for suitably stabilized formulations, while maintenance of a sufficiently high viable-cell concentration throughout storage is essential for effective delivery and subsequent colonization [89]. For solid carrier formulations, moisture content is particularly important because excessive water activity accelerates microbial metabolism and loss of stability; moisture contents of approximately 10–20% have commonly been used or recommended for dried carrier-based inoculants, although the optimum value is carrier- and microorganism-specific [89,90]. Storage temperature is similarly formulation-dependent: refrigeration at approximately 4 °C can improve survival of sensitive non-sporulating bacteria, whereas commercially robust formulations are preferably designed to retain acceptable viability under ambient storage conditions [89]. Application dose cannot be standardized solely according to formulation class because effective doses depend on viable-cell density, microbial strain, crop, delivery method, and target colonization site. Likewise, field persistence may range from transient establishment to persistence over substantial portions of the crop cycle and should therefore be quantified using strain-specific culture-dependent or molecular tracking approaches rather than assumed from the formulation type [89,91]. Representative quantitative benchmarks and reported ranges for the major formulation and delivery systems are summarized in Table 4. Granular formulations generally provide greater storage stability and prolonged release, whereas liquid formulations facilitate rapid and uniform application. Seed coatings promote early root colonization after germination, while encapsulation systems protect microbial cells against desiccation, ultraviolet radiation, and temperature fluctuations, thereby improving their survival and persistence after application [89,92].

Carrier materials play a fundamental role in determining formulation quality and microbial performance. Suitable carriers should provide physical protection, maintain moisture availability, support microbial metabolism, and ensure gradual release of viable microorganisms into the rhizosphere. Organic carriers such as peat, compost, lignite, vermiculite, and biochar, together with synthetic polymers and biodegradable hydrogels, have demonstrated considerable potential for improving microbial survival and colonization efficiency. Furthermore, advances in nanotechnology and smart carrier materials are opening new opportunities to develop controlled-release formulations that deliver microorganisms in a targeted and environmentally responsive manner [92]. The method of application also substantially influences microbial establishment and field performance. Microbial biostimulants can be delivered through seed treatment or coating, root dipping, soil application or drenching, fertigation, and foliar spraying, with each approach presenting specific advantages and limitations depending on the crop species, developmental stage, microbial formulation, environmental conditions, and production system [93,94]. Consequently, selection of an appropriate delivery strategy requires consideration of microbial characteristics, crop management practices, application equipment, soil conditions, and compatibility with pesticides and other agricultural inputs. Optimizing formulation, application timing, dose, and delivery method is therefore essential for maximizing microbial survival, establishment, and functional performance under field conditions [94]. Despite significant technological advances, several challenges continue to limit the widespread adoption of microbial biostimulants. Maintaining long-term shelf life, preserving microbial viability during storage, ensuring compatibility among microorganisms within microbial consortia, preventing contamination, and achieving consistent field performance remain major priorities for commercial product development. In addition, formulation standardization, quality assurance, regulatory compliance, and cost-effective large-scale manufacturing are essential prerequisites for successful commercialization. Continued innovation in formulation science, carrier technology, and precision delivery systems will therefore be critical for maximizing the practical value of microbial biostimulants in sustainable agriculture [89]. Overall, advances in formulation technologies and delivery systems are expected to play a decisive role in the future success of microbial biostimulants. The integration of stable formulations, intelligent carrier materials, precision application technologies, and, where supported by comparative evidence, microbial consortia is expected to improve product reliability, enhance field persistence, and increase adoption by farmers. Such technological developments will facilitate the broader implementation of microbial biostimulants as key components of climate-smart and sustainable agricultural production systems [95]. The principal formulation technologies currently used for microbial biostimulants, together with their advantages, limitations, and major agricultural applications, are summarized in Table 4.

**Table 4:** Comparison of formulation technologies and quantitative performance parameters reported for microbial biostimulants.

Formulation Type	Typical Carrier/Material	Viability/Viable-Cell Density	Shelf Life and Storage	Moisture/Stability Considerations	Application/Dose Considerations	Field Persistence/Major Feature	Ref.
Liquid suspension	Water- or oil-based media; protective polymers/additives	Commonly formulated at high viable-cell densities; the required CFU mL^−1^ is strain- and product-specific; reported examples include 10^9^–10^10^ CFU mL^−1^ declared for a commercial *Pseudomonas chlororaphis* product and approximately 2 × 10^9^ cells mL^−1^ for common liquid inoculants	Shelf life ranges from several months to ≥1 year in optimized formulations; temperature, oxygen and packaging strongly affect survival; reported examples range from 3 weeks at room temperature (8 weeks at 4–8 °C) for a non-sporulating *Pseudomonas* product to up to 2 years for cool-stored liquid inoculants	Water activity and formulation additives influence stability; no universal moisture benchmark applies to liquids	Applied by seed treatment, root dipping, fertigation or foliar spraying; dose should be expressed as viable cells delivered per seed, plant, or unit area where possible; for *Azospirillum brasilense*, approximately 10^6^–10^7^ cells plant^−1^ has been reported as the threshold required for a positive plant response	Rapid and uniform delivery, but persistence after application depends strongly on strain, crop and environment; up to 90% of introduced cells may be lost during field application, and inoculant populations are often transient, declining within weeks to months	[89,90,94]
Wettable powder/dry carrier	Talc, peat, lignite, starch and related carriers	Viability should be monitored as CFU g^−1^ throughout storage; acceptable density is organism- and product-specific; commercial carrier-based rhizobial products are typically declared at approximately 3 × 10^8^–1 × 10^9^ CFU g^−1^	Often provides greater storage stability than unprotected cells; shelf life can extend from months to >1 year under optimized conditions; for common solid carrier-based inoculants, approximately 6 months, reaching 12–18 months in the best cases	Residual moisture is critical; approximately 10–20% has been reported/recommended for some carrier systems, but optimum values are carrier-specific	Reconstituted before application or used for seed/soil treatment; application rate depends on viable count and crop; e.g., peat inoculants containing ~10^9^ rhizobia g^−1^ applied at ~250 g ha^−1^ deliver ~2.5 × 10^10^ cells ha^−1^	Drying improves storage stability but can reduce viability of desiccation-sensitive microorganisms; formulation-specific quantitative field-persistence data were not identified in the cited literature	[89,90,94]
Granular formulation	Clay, peat, compost, vermiculite, biochar	Viability commonly quantified as CFU g^−1^; formulation must retain an effective population during storage and after soil incorporation; no representative formulation-specific CFU g^−1^ benchmark was identified in the cited literature	Generally suitable for extended storage when moisture and temperature are controlled	Low, controlled moisture reduces metabolic activity; exact optimum depends on carrier and organism	Usually soil- or furrow-applied; higher mass application rates than seed coatings are generally required; reliable quantitative dose benchmarks were not identified in the cited literature	Gradual release can extend microbial availability near the root zone, although actual persistence requires strain-specific verification	[89,90]
Seed coating	Polymers, alginate, starch, cellulose derivatives	Viability should preferably be reported as viable cells per seed and monitored during seed storage; for *A. brasilense*, ~10^6^–10^7^ cells plant^−1^ has been reported as a response threshold	Survival may decline during storage depending on seed species, coating chemistry and temperature; e.g., a biochar-based coating maintained high *Bradyrhizobium japonicum* populations for >4 months, and lyophilized alginate beads coated onto seeds retained viability for ≥3 months at ambient temperature	Water activity is important; high rhizobial survival on coated seeds has been reported at aᵥ ≈ 0.38–0.47	Low inoculum volume with direct delivery to emerging roots; dose is best expressed as viable cells seed^−1^; in maize, seed coating with ~270 AMF propagules seed^−1^ achieved root colonization similar to soil inoculation with ~4900 propagules plant^−1^	Favors early rhizosphere colonization, but persistence beyond establishment is highly strain- and environment-dependent	[89,90,93]
Encapsulation	Alginate, chitosan, carrageenan, hydrogels	High initial cell loading can be protected within beads/capsules; viable counts should be monitored during storage and release; reported loadings range from 10^8^–10^9^ CFU g^−1^ beads (e.g., *A. brasilense* and *Pseudomonas fluorescens*) to up to 10^9^ CFU bead^−1^	Can extend survival relative to unprotected cells by buffering desiccation and environmental stress; e.g., survival for ≥12 months at room temperature in chitosan–starch beads and full recovery of viable *P. fluorescens* after 250 days in skim milk–alginate beads	Polymer composition, bead water content and drying conditions determine stability	Applied to seed/soil/root zone; dose depends on cell loading per bead/capsule and number delivered	Controlled release may prolong local survival and colonization; e.g., encapsulated *Pseudomonas putida* Rs-198 increased from 2.1 × 10^4^ to 9.2 × 10^5^ CFU g^−1^ soil over 21 days; persistence remains dependent on soil and host conditions	[89,91]
Biochar-based formulation	Biochar and biochar composites	CFU g^−1^ depends on microbial strain, pore structure and loading procedure; no representative benchmark was identified in the cited literature	Potentially supports prolonged survival (e.g., >4 months for *B. japonicum* in a biochar-based seed coating), but storage performance varies with feedstock and production conditions	Moisture retention and porous structure can protect microorganisms from desiccation	Mainly incorporated into soil; dose depends on both microbial loading and biochar application rate; reliable quantitative dose benchmarks were not identified	May provide a persistent physical microsite for microbial establishment while also improving soil water retention	[90,92,93]
Nanocarrier-based formulation	Silica nanoparticles, nanoclays, nanopolymers and nano-enabled composites	No broadly accepted viable-cell-density benchmark is established across nano-enabled microbial formulations	Potential for enhanced protection and controlled release, but standardized shelf-life benchmarks remain limited	Stability depends on carrier chemistry, water activity and microorganism–nanomaterial compatibility	Application rates remain formulation-specific and require efficacy and biosafety validation	Controlled/targeted delivery is promising, but robust quantitative evidence for long-term field persistence remains limited	[91,92]

The quantitative parameters summarized in Table 4 should be interpreted as formulation-development benchmarks or reported ranges rather than universal specifications. Direct comparison among formulations remains difficult because studies differ in microbial strain, carrier composition, viability assay, storage conditions, application rate, crop, soil environment, and the method and duration used to assess post-application persistence [89,90,91]. Standardized reporting of initial and end-of-storage viable-cell density, storage temperature and duration, carrier moisture or water activity, application dose expressed on a viable-cell basis, and strain-specific persistence after application would substantially improve comparability among microbial biostimulant formulations. Where representative numerical values were not identified in the cited literature, particularly for viable-cell density and application dose of granular, biochar-based and nanocarrier-based formulations and for formulation-specific field persistence, this limitation is indicated in Table 4, and the values reported for individual products or strains should not be extrapolated to other microorganisms or formulations.

### 4.3. Environmental and Economic Considerations

Addressing the dual challenges of climate change and global food security requires agricultural systems that simultaneously increase productivity while minimizing environmental impacts. Modern agriculture depends heavily on synthetic fertilizers, pesticides, and intensive irrigation, all of which contribute substantially to greenhouse gas emissions, soil degradation, biodiversity loss, and freshwater depletion. In particular, excessive nitrogen fertilization contributes significantly to nitrous oxide (N_2_O) emissions, whereas intensive crop production systems accelerate carbon losses from agricultural soils [96,97]. Consequently, developing sustainable alternatives that reduce external agricultural inputs while maintaining crop productivity has become a global priority. Beyond their potential environmental benefits, microbial biostimulants may also improve the economic performance of crop production, although profitability is strongly dependent on crop value, yield response, inoculant cost, fertilizer and irrigation inputs, and local input and output prices. Quantitative field studies illustrate this context dependence. In a two-year potato field experiment, a liquid phosphate-solubilizing bacterial formulation increased tuber yield by an average of 16.2% and generated an additional mean net return of INR 44,876 ha^−1^ relative to the corresponding uninoculated control; the reported benefit–cost ratio increased by approximately 0.40, reaching 2.52 in the inoculated treatment [98]. In garden pea, Rhizobium inoculation increased gross return from USD 2927 to USD 3460 ha^−1^ for green-pod production, while the corresponding benefit–cost ratio increased from 5.07 to 5.70; however, combinations of inoculation with comparatively expensive micronutrient treatments did not necessarily produce higher benefit–cost ratios [99]. Similarly, field studies in other crops have reported favorable economic responses to microbial inoculation, but the magnitude of these benefits varies substantially with the production system and the costs included in the analysis [99,100]. These findings indicate that microbial inoculation can improve profitability when additional yield or input-use efficiency exceeds the cost of the microbial treatment, but favorable economic returns should not be generalized across crops or agroecosystems without system-specific economic evaluation.

**Figure 7:**
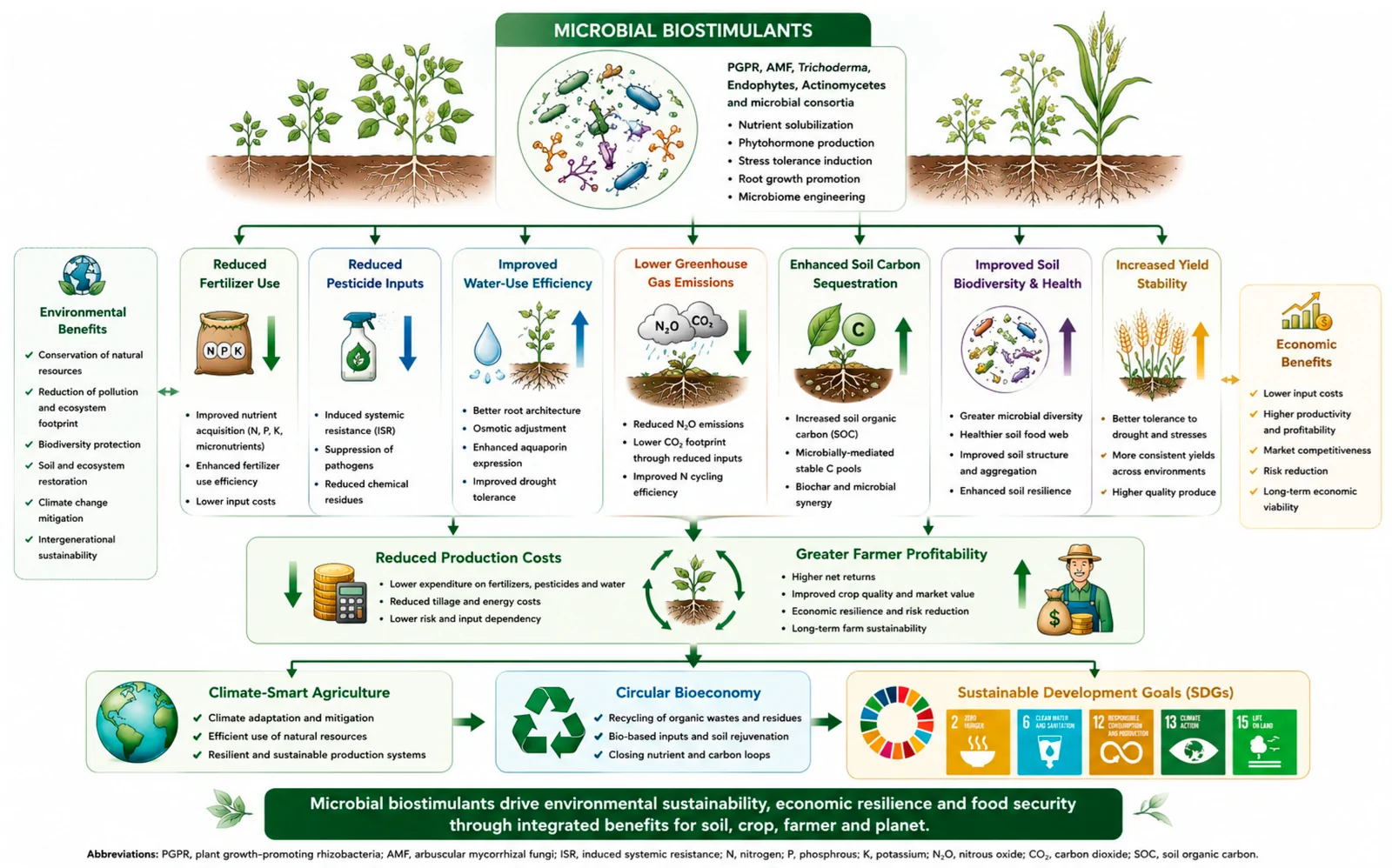
Environmental and economic benefits of microbial biostimulants for sustainable and climate-smart agriculture. Microbial biostimulants, including plant growth-promoting rhizobacteria (PGPR), arbuscular mycorrhizal fungi (AMF), *Trichoderma* spp., endophytes, actinomycetes, and microbial consortia, contribute to sustainable crop production through multiple interconnected environmental and economic benefits. Their application improves nutrient acquisition and fertilizer use efficiency, reduces pesticide dependence, enhances water use efficiency, lowers greenhouse gas emissions, promotes soil carbon sequestration, increases soil biodiversity and ecosystem resilience, and improves crop productivity and yield stability under drought conditions. These improvements reduce production costs, increase farm profitability, strengthen economic resilience, and support sustainable farming systems. Collectively, microbial biostimulants contribute to climate-smart agriculture, circular bioeconomy strategies, efficient resource utilization, and several United Nations Sustainable Development Goals (SDGs), thereby promoting long-term food security and environmental sustainability under changing climatic conditions. In the figure, arrows indicate the directional relationships and interconnected effects among microbial biostimulant application, environmental benefits, economic outcomes, and broader sustainability goals, while check marks (✓) indicate beneficial or desirable outcomes associated with microbial biostimulant use.. Abbreviations: PGPR, plant growth-promoting rhizobacteria; AMF, arbuscular mycorrhizal fungi; ISR, induced systemic resistance; N, nitrogen; P, phosphorus; K, potassium; N_2_O, nitrous oxide; CO_2_, carbon dioxide; SOC, soil organic carbon; SDGs, Sustainable Development Goals.

Despite this economic potential, the widespread adoption of microbial biostimulants remains constrained by several socioeconomic factors. Many farmers, particularly smallholder producers and those operating with limited financial resources, face difficulties accessing innovative microbial technologies due to high product costs, limited technical knowledge, inadequate extension services, and uncertainty about field performance under diverse environmental conditions. Furthermore, inconsistent regulatory frameworks, limited commercialization infrastructure, and variable product quality continue to restrict market expansion in many countries [64]. The successful implementation of microbial biostimulants, therefore, requires an integrated approach that combines scientific innovation with appropriate regulatory frameworks, farmer awareness, and supportive agricultural policies. Policies encouraging sustainable production, improved resource-use efficiency, and reduced dependence on chemical inputs could facilitate wider adoption of microbial technologies. Furthermore, harmonized regulatory frameworks, standardized quality-control procedures, and transparent efficacy assessment are essential for increasing stakeholder confidence and facilitating the commercialization of microbial biostimulant products [101]. Microbial biostimulants are also consistent with broader principles of climate-smart and circular agricultural systems, in which biological resources are utilized to improve resource-use efficiency, reduce environmental impacts, and strengthen agroecosystem resilience. By promoting biological nutrient cycling, reducing reliance on agrochemical inputs, enhancing soil health, and increasing plant resilience to environmental stresses, microbial inoculants can contribute to agricultural sustainability and climate change adaptation while potentially reducing the environmental footprint of crop production [38,102]. Their integration into precision, conservation, and regenerative agricultural systems therefore represents a promising pathway toward achieving several United Nations Sustainable Development Goals (SDGs), particularly Zero Hunger (SDG 2), Responsible Consumption and Production (SDG 12), Climate Action (SDG 13), and Life on Land (SDG 15) [102]. Overall, microbial biostimulants provide a promising opportunity to reconcile agricultural productivity with environmental sustainability. Although challenges related to field consistency, commercialization, farmer adoption, product quality, and regulatory harmonization remain, continued advances in microbial biotechnology, formulation science, microbiome engineering, and precision agriculture are expected to improve the reliability and practical viability of microbial biostimulants [94]. Consequently, these biological products have considerable potential to become important components of future sustainable and climate-resilient agricultural systems. Their environmental and economic benefits may extend beyond improvements in crop productivity through enhanced resource-use efficiency, reduced dependence on conventional agricultural inputs, improved soil functioning, and greater resilience of agricultural production systems [94]. These integrated benefits are summarized in Figure 7, which illustrates the multiple pathways through which microbial biostimulants support climate-smart agriculture, circular bioeconomy principles, and global sustainable development goals.

### 4.4. Regulation, Safety, and Quality Assurance Aspects

The successful commercialization and widespread adoption of microbial biostimulants depend not only on their biological efficacy but also on robust regulatory frameworks, comprehensive biosafety assessments, and rigorous quality assurance procedures. Although numerous studies have demonstrated the capacity of microbial biostimulants to enhance drought tolerance and improve crop productivity under water-limited conditions, their transition from experimental research to commercial agricultural products requires standardized regulations that ensure product safety, efficacy, consistency, and environmental sustainability [103]. Microbial biostimulants, including plant growth-promoting rhizobacteria (PGPR), arbuscular mycorrhizal fungi (AMF), *Trichoderma* spp., and other beneficial microorganisms, are generally regarded as environmentally safe alternatives to conventional agrochemicals because they naturally occur within agricultural ecosystems and improve plant performance through biological processes rather than chemical inputs. Nevertheless, commercial microbial products must undergo rigorous evaluation to confirm strain identity, genetic stability, ecological safety, absence of pathogenicity, and compatibility with native microbial communities before large-scale agricultural application. These biosafety evaluations are particularly important to ensure that introduced microorganisms do not adversely affect non-target organisms or disrupt indigenous soil microbial diversity [104]. Regulation (EU) 2019/1009 establishes requirements concerning safety, labeling, demonstrated efficacy, and conformity assessment for products placed on the EU market [105]. However, the current EU framework for microbial plant biostimulants is restricted to microorganisms listed under Component Material Category 7 (CMC 7), namely *Azotobacter* spp., mycorrhizal fungi, *Rhizobium* spp., and *Azospirillum* spp., leaving many potentially valuable microbial taxa outside this harmonized pathway [105,106]. Regulatory approaches outside the European Union differ substantially in product classification, registration requirements, safety assessment, and efficacy documentation. In Canada, microbial inoculants are regulated as microbial supplements under the federal Fertilizers Act and Fertilizers Regulations, with the Canadian Food Inspection Agency (CFIA) requiring pre-market registration for microbial supplements and information on microbial identity, composition, safety, and labeling [107]. In India, biostimulants, including microorganisms or combinations of substances and microorganisms that stimulate plant physiological processes, are regulated under the Fertilizer (Control) Order (FCO), 1985, following their formal inclusion in the FCO regulatory framework in 2021; biofertilizer categories are likewise subject to specified quality standards [108]. Brazil has recently established a broader national framework for agricultural bioinputs through Law No. 15,070/2024, covering production, registration, commercialization, use, inspection, labeling, storage, and other aspects of bioinputs, including biofertilizers, inoculants, and biostimulants [109]. These approaches differ from the EU framework in regulatory scope, product categorization, eligible microorganisms, and pre-market requirements, illustrating why international commercialization remains complex. A concise comparison of representative regulatory pathways is provided in Table 5.

The comparison in Table 5 highlights substantial differences among jurisdictions in product classification, eligible microorganisms, registration procedures, safety assessment, quality requirements, and the evidence required for market authorization. Such regulatory heterogeneity can complicate international product development and commercialization and reinforces the need for greater convergence in terminology, microbial identification and quality-control standards, safety assessment, efficacy documentation, and labeling requirements [105,106,107,108,109,110,111]. Commercial formulations must maintain consistent microbial viability, purity, genetic identity, shelf life, and functional activity throughout production, storage, transportation, and field application. Standardized manufacturing protocols, molecular authentication of microbial strains, contamination monitoring, viability testing, and batch-to-batch consistency are therefore essential components of quality-control systems. Moreover, formulation technologies should preserve microbial survival while ensuring compatibility with fertilizers, pesticides, irrigation systems, and other agricultural inputs commonly used in modern crop production [89]. Beyond regulatory compliance, successful adoption of microbial biostimulants also requires transparent product labeling, standardized efficacy assessment, reliable quality control, farmer education, and appropriate post-market surveillance. Greater international harmonization of regulatory frameworks could facilitate commercialization, simplify product registration, and promote wider adoption of microbial technologies in sustainable and climate-resilient agriculture [110,111]. Continued collaboration among researchers, industry, policymakers, and regulatory authorities will therefore be essential for developing science-based standards that promote innovation while ensuring product quality, efficacy, biosafety, and environmental sustainability. Despite considerable progress in microbial biostimulant research and commercialization, scientific, technological, regulatory, and socioeconomic challenges continue to constrain the consistent and widespread adoption of microbial biostimulants in agriculture [110,111]. Future research and commercial development should therefore focus on the following priorities:

- Development of standardized regulatory frameworks and international harmonization of product registration procedures.
- Improvement of formulation technologies to enhance microbial viability, shelf life, and field persistence.
- Establishment of internationally accepted quality-control standards for microbial identification, purity, efficacy, and batch-to-batch consistency.
- Long-term, multi-location field validation across different crop species, soil types, climatic conditions, and irrigation regimes.
- Development of synthetic microbial communities (SynComs) with complementary physiological functions and improved ecological stability.
- Integration of multi-omics technologies, systems biology, and microbiome engineering to optimize microbial selection and formulation.
- Application of artificial intelligence and machine learning approaches to predict plant–microbe compatibility and improve inoculant performance under diverse agroecosystems.
- Integration of microbial biostimulants with precision agriculture, climate-smart agriculture, integrated pest management (IPM), and sustainable nutrient management programs.
- Expansion of farmer education, extension services, and technology-transfer programs to improve awareness and adoption of microbial technologies.
- Continuous post-commercialization monitoring to evaluate long-term ecological safety, environmental impacts, and product effectiveness under practical farming conditions.

Addressing these priorities will substantially improve the reliability, scalability, and commercial success of microbial biostimulants while accelerating their integration into sustainable and climate-resilient agricultural systems. The current state of microbial biostimulant development, major implementation challenges, and future opportunities for drought-resilient agriculture are summarized in Table 6.

## 5. Knowledge Gaps, Future Directions, and Research Priorities

Despite substantial progress, a major unresolved question is why microbial biostimulants that perform effectively under controlled conditions often show variable responses in the field. Although plant genotype, soil properties, indigenous microbiota, drought intensity, and management practices are recognized as important determinants, their relative contributions and interactions remain poorly resolved. Future research should therefore prioritize standardized, long-term, multi-location experiments designed to identify reproducible predictors of microbial establishment, persistence, and drought-mitigation efficacy across contrasting agroecosystems [112,113]. A second critical gap concerns causality in plant–microbe responses to drought. Many studies report associations between microbial inoculation and changes in gene expression, metabolites, phytohormones, antioxidant activity, or microbiome composition; however, the microbial traits and plant responses directly responsible for improved drought resilience are often difficult to distinguish from secondary responses. Future studies should therefore combine complementary molecular approaches with targeted functional validation to identify causal microbial functions, host regulatory pathways, and interaction mechanisms that determine successful drought adaptation [45].

Finally, improved cross-study comparability will require consistent reporting of key experimental variables, including microbial strain identity and inoculum viability or concentration, inoculation method and timing, plant species and genotype, developmental stage at treatment, growth substrate and soil physicochemical properties, drought-imposition method, duration and severity of water deficit, soil moisture or water-potential measurements, environmental conditions, and the timing of physiological and agronomic assessments. Studies should also clearly define drought-response endpoints, including relative water content, gas-exchange parameters, biomass, yield, and water-use efficiency when directly measured, together with the corresponding well-watered and uninoculated controls. Consistent reporting of these variables would improve reproducibility, facilitate meaningful comparisons among studies, and provide a stronger basis for evaluating microbial establishment, persistence, and drought-mitigation efficacy across crops and environments [113].

## 6. Conclusions

Developing sustainable strategies to improve crop drought resilience is increasingly important in the context of climate change, water scarcity, and growing demands for agricultural production. The evidence reviewed here indicates that microbial biostimulants have considerable potential to support plant adaptation to water deficit through interconnected physiological, biochemical, molecular, and ecological processes, including improved water and nutrient acquisition, modulation of phytohormone signaling, osmotic adjustment, antioxidant defense, and interactions with the plant-associated microbiome. However, the magnitude and consistency of these responses vary substantially among microbial strains and consortia, plant species and genotypes, soil properties, indigenous microbial communities, drought regimes, climatic conditions, and crop management practices. Positive responses obtained under controlled conditions therefore cannot be assumed to translate directly into consistent agronomic benefits under field conditions. Available field evidence supports the potential of microbial biostimulants to improve plant performance and, in some crop–microbe–environment combinations, yield or yield stability under water-limited conditions. Nevertheless, neutral, variable, or management-dependent responses have also been reported, emphasizing that microbial biostimulants should be considered context-dependent components of integrated drought-management strategies rather than universally effective standalone solutions. Improving their reliability will require standardized experimental and reporting frameworks, quantitative assessment of microbial establishment and persistence, long-term and multi-location field validation, robust formulation and quality-control procedures, and better characterization of interactions among plant genotype, soil, microbiome, environment, and management.

Future progress is likely to depend increasingly on systems-level approaches that extend beyond empirical selection of individual microbial strains. Synthetic microbial communities (SynComs), microbiome engineering, improved formulation and delivery technologies, multi-omics approaches, predictive modeling, and precision agriculture provide opportunities to identify and deploy microbial functions more effectively within defined agroecosystems. However, these emerging approaches require rigorous validation to determine whether improved functional complementarity and microbial persistence translate into reproducible agronomic benefits under variable field conditions. Overall, microbial biostimulants represent a promising component of climate-resilient and sustainable crop management, but realizing their full agricultural potential will depend on converting mechanistic and controlled-environment evidence into reliable, context-specific, and economically viable field applications.

## Figures and Tables

**Table 2:** Operational variables and decision criteria for applying the G × S × M × E framework to microbial biostimulant selection under drought.

Framework Component	Measurable Variables	Examples of Measurement	Proposed Decision Criterion
Genotype (G)	Crop/cultivar; root architecture; phenology; drought sensitivity; physiological response to inoculation	Root length/biomass; phenological stage; RWC; gas exchange; biomass/yield	Retain inoculants showing reproducible beneficial responses in the target genotype relative to the appropriate uninoculated control
Soil (S)	pH; texture; organic matter; EC; nutrient status; water-holding capacity; baseline moisture	Standard soil analyses; gravimetric/VWC measurements; water-retention characteristics	Validate efficacy across the soil properties representative of the intended production environment; exclude combinations in which establishment or benefit is consistently lost
Microbiome (M)	Indigenous community composition/diversity; functional taxa/genes; inoculant establishment and persistence; compatibility with resident microbiota	Amplicon sequencing/metagenomics; strain-specific qPCR or other molecular tracking; colonization assays	Require detectable establishment/persistence and absence of reproducible antagonistic effects; prioritize combinations in which inoculation produces consistent functional benefit
Environment (E)	Drought intensity, duration, timing and frequency; soil water status; temperature; precipitation; evaporative demand; irrigation regime	Soil water potential/VWC; meteorological data; irrigation records	Evaluate inoculants under drought regimes representative of the target environment and require reproducible benefit across predefined stress scenarios
Integrated G × S × M × E performance	Establishment plus plant physiological/agronomic response across combinations	Factorial experiments; multi-location trials; mixed models/multivariate or predictive modeling	Advance candidates only when establishment and predefined drought-response benefits are reproducible across representative combinations and are statistically supported relative to appropriate controls

**Table 3:** Representative field trials evaluating microbial inoculants under water-limited conditions and their effects on crop yield.

Crop	Microbial Inoculant	Location	Drought/Irrigation Treatment	Yield Response	Statistical Outcome	Ref.
Tomato (*Solanum lycopersicum* L.)	*Azotobacter chroococcum* 76A + *Trichoderma afroharzianum* T22	Bellizzi, Italy; experimental farm, University of Naples Federico II	Optimal input vs. 70% of optimal water supply combined with 70% N input	Across input levels, inoculation increased mean yield from 2410 to 3110 g plant^−1^. Under optimal inputs, yield increased by 48.6%; under combined water/N limitation, the consortium did not compensate for the stress-induced yield reduction.	Inoculant treatment significantly affected yield (*p* < 0.05); input × inoculant interaction was also significant (*p* < 0.05). Yield improvement under suboptimal water/N conditions was not sufficient to overcome the stress effect.	[6]
Wheat (*Triticum aestivum* L.)	Consortium of *Azotobacter*, *Azospirillum*, *Microbacterium*, and *Pseudomonas* spp.	Agricultural and Natural Resources Research Center, Hamedan, Iran	Two-year field trial; normal, moderate, and severe drought stress	Inoculation increased grain yield by approximately 10% overall; the greatest increase occurred under moderate drought (~12%).	Grain yield was significantly increased by inoculation under normal and moderate stress; under severe drought, the inoculant did not significantly affect grain yield.	[79]
Wheat (*Triticum aestivum* L.; cvs. Jimai 22 and Chang 6878)	*Streptomyces pactum* Act12	Hancun Experimental Station, Wheat Research Institute, Shanxi Agricultural University, Shanxi, China	Field drought conditions during the 2018–2019 growing season; drought responses evaluated at jointing, heading, and grain-filling stages	Under drought, Act12 increased yield by 11.78% in drought-sensitive Jimai 22 and 13.55% in drought-resistant Chang 6878.	Act12 significantly improved physiological and yield-related responses under drought; treatment effects were genotype-dependent.	[80]
Cucumber (*Cucumis sativus* L.)	AMF alone and AMF + PGPR co-inoculation	Field experiment, Eskişehir, Türkiye	Two growing seasons; four irrigation levels: 33%, 66%, 100%, and 133% irrigation	Under the most severe deficit treatment (33%), AMF and AMF + PGPR increased yield by 66.52% and 33.93%, respectively, relative to uninoculated plants; microbial treatments also mitigated >40% yield loss observed in uninoculated plants.	Microbial-biostimulant effects on yield and WUE depended on irrigation level; AMF alone produced the strongest yield response under severe water deficit.	[81]
Soybean (*Glycine max* L. cv. Giza 111)	Drought-tolerant *Bradyrhizobium japonicum* DTB4 and *Bacillus subtilis* DTR30, alone or combined with AMF (*Glomus clarum*, *Funneliformis mosseae*, *Gigaspora margarita*)	Agronomy Department Farm, Mansoura University, Egypt	Irrigation withheld at critical R3 (early pod) or R5 (seed-development) stages	Bioinoculation increased pods plant^−1^, 100-seed weight, and grain yield under drought; combined microbial treatments produced some of the strongest responses.	Bioinoculant treatments significantly improved yield components and grain yield under drought (*p* ≤ 0.05); effects depended on drought timing and inoculant combination.	[82]
Bread wheat (*Triticum aestivum* L.)	Synthetic consortium including *Azospirillum argentinensis* Az39, *Scenedesmus obliquus* C1S and associated bacterial component(s)	INTA Balcarce Experimental Station, Buenos Aires, Argentina	Rainfed field trials in 2022 and 2024 under environmental drought; agroecological, no-till, and conventional management systems	Triple consortium increased grain yield from 1927 to 2244 kg ha^−1^ under agroecological management in 2022 and from 3046 to 4130 kg ha^−1^ under conventional management in 2024 (~36% in 2024).	Yield increases were significant under agroecological and conventional management, but the positive response was absent under no-till management, demonstrating management dependence.	[83]
Soybean (*Glycine max* L.)	*Bacillus subtilis* 1A11 and *Bacillus velezensis* 5D5 and 6E9	Birigui, Itapira, and Piracicaba, São Paulo State, Brazil	Multi-location field trials during 2022–2023 and 2023–2024; natural climatic variability, including high temperature and reduced rainfall in 2023–2024	Inoculated treatments improved grain yield by approximately 11.3–18%; *B. subtilis* 1A11 produced the largest reported increase, up to 620 kg ha^−1^ over the control.	Statistical modeling indicated significant positive effects of the inoculants on soybean development and grain yield; magnitude varied by location and season.	[84]

**Table 5:** Comparison of representative regulatory pathways relevant to microbial biostimulants and microbial inoculants.

Region/Country	Principal Regulatory Framework	Regulatory Approach	Key Features Relevant to Microbial Products	Ref.
European Union	Regulation (EU) 2019/1009	EU fertilizing product/plant biostimulant	Harmonized EU pathway; microbial plant biostimulants under CMC 7 are presently restricted to *Azotobacter* spp., mycorrhizal fungi, *Rhizobium* spp., and *Azospirillum* spp.; conformity, safety and labeling requirements apply	[105,106]
Canada	Fertilizers Act and Fertilizers Regulations	Fertilizer or supplement; microbial inoculants generally regulated as microbial supplements	CFIA pre-market registration applies to microbial supplements; assessment includes taxonomic identity, composition, safety/hazard information and labeling; microbial consortia are specifically addressed	[107]
India	Fertiliser (Control) Order, 1985, as amended	Biofertilizer/biostimulant framework	Biostimulants were formally incorporated into the FCO framework in 2021; the definition includes microorganisms and microorganism-containing combinations, while biofertilizer categories are subject to quality specifications	[108]
Brazil	Law No. 15,070/2024 (Bioinputs Law)	Broad bioinputs framework	Covers biofertilizers, inoculants, biostimulants and other agricultural bioinputs and regulates production, import/export, registration, commercialization, use, inspection, labeling, transport and storage	[110]

**Table 6:** Current status and prospects of microbial biostimulants for drought-resilient agriculture.

Aspect	Current Status	Major Challenges	Future Opportunities	References
Field efficacy	Positive responses were demonstrated in cereals, legumes, vegetables, fruit crops, and horticultural species under greenhouse and field conditions.	High variability among environments, crop species, soil types, and climatic conditions.	Precision selection of microbial inoculants based on crop genotype, soil characteristics, and environmental conditions.	[2,17,75,78]
Microbial diversity	PGPR, AMF, *Trichoderma*, endophytes, and microbial consortia are increasingly used.	Inconsistent establishment and competition with indigenous microbiota.	Development of synthetic microbial communities (SynComs) and microbiome engineering strategies.	[45,51,61]
Formulation technologies	Commercial products are available as liquid suspensions, wettable powders, granules, seed coatings, and soil amendments.	Limited shelf life, reduced microbial viability during storage, formulation incompatibility, and inconsistent field persistence.	Encapsulation technologies, nanocarriers, controlled-release formulations, and stress-protective carriers.	[89,92,93]
Application methods	Seed treatment, root dipping, soil application, fertigation, and foliar application are widely adopted.	Crop- and environment-specific optimization remains limited.	Precision agriculture, smart delivery systems, and site-specific application strategies.	[93,94]
Mechanistic understanding	Considerable advances have been made in understanding phytohormone regulation, antioxidant defense, nutrient acquisition, and microbiome assembly.	Complex interactions among plants, microorganisms, soil, and climate remain incompletely understood.	Systems biology, multi-omics integration, network biology, and predictive modeling.	[17,22,45,69]
Omics technologies	Increasing application of genomics, transcriptomics, proteomics, metabolomics, and metagenomics.	High analytical cost, large datasets, and limited integration across studies.	Artificial intelligence (AI), machine learning, digital agriculture, and predictive microbiome engineering.	[23,55,69]
Regulatory framework	Regulatory frameworks for microbial biostimulants and related microbial products differ substantially among jurisdictions, as summarized in Table 5.	Differences in product classification, eligible microorganisms, registration procedures, efficacy requirements, and quality standards among jurisdictions.	Greater international convergence in terminology, safety assessment, efficacy testing, microbial identification, quality control, and labeling requirements.	[105,106,107,108,109,110,111]
Safety and quality assurance	Increasing emphasis on biosafety assessment, strain identification, and quality control during production.	Variation in product quality, contamination risks, and limited post-commercial monitoring.	International certification systems, molecular quality control, and standardized manufacturing protocols.	[89,110,111]
Integration into agricultural systems	Increasing use within integrated nutrient management (INM), integrated pest management (IPM), and climate-smart agriculture.	Farmer awareness, economic uncertainty, and inconsistent performance under diverse farming systems.	Climate-resilient cropping systems, regenerative agriculture, carbon farming, and precision microbial management.	[38,86,87,103]
Future research priorities	Growing interest in multidisciplinary research linking microbiology, plant physiology, soil science, and biotechnology.	Limited long-term multi-location validation and insufficient understanding of context dependency.	Large-scale field validation, G × S × M × E modeling, customized microbial consortia, and next-generation biostimulant products.	[45,69,70,110]

## Data Availability

No new data were created or analyzed in this study. Data sharing is not applicable to this article.

## Abbreviations

ABA, abscisic acid; ACC, 1-aminocyclopropane-1-carboxylate; AI, artificial intelligence; AMF, arbuscular mycorrhizal fungi; APX, ascorbate peroxidase; BRs, brassinosteroids; CAT, catalase; CK, cytokinins; DREB, dehydration-responsive element-binding protein; EC, electrical conductivity; EPS, extracellular polymeric substances; ET, ethylene; GA, gibberellins; IAA, indole-3-acetic acid; IPM, integrated pest management; JA, jasmonic acid; MAPK, mitogen-activated protein kinase; NO, nitric oxide; NPK, nitrogen–phosphorus–potassium; PGPR, plant growth-promoting rhizobacteria; PIPs, plasma membrane intrinsic proteins; POD, peroxidase; ROS, reactive oxygen species; RWC, relative water content; SA, salicylic acid; SLs, strigolactones; SOD, superoxide dismutase; SynComs, synthetic microbial communities; TIPs, tonoplast intrinsic proteins; VOCs, volatile organic compounds; WUE, water-use efficiency.
